# Glycemic and bodyweight effects of *GIPR* coding variation reflect differences in surface expression and intrinsic functional impairment

**DOI:** 10.1126/sciadv.aec3372

**Published:** 2026-09-04

**Authors:** Yusman Manchanda, Rofaida Desoki, Eugene J. Gardner, John R. B. Perry, David B. Wainscott, Cynthia Stutsman, Claudia Langenberg, Matthew Coghlan, Pallav Bhatnagar, Joseph D. Ho, Michael Yonkunas, Kyle W. Sloop, Ken K. Ong, Nicholas J. Wareham, Alejandra Tomas, Ben Jones

**Affiliations:** ^1^ Section of Cell Biology and Functional Genomics, Department of Metabolism, Digestion and Reproduction, Faculty of Medicine, Imperial College London, London W12 0NN, UK.; ^2^ MRC Epidemiology Unit, Institute of Metabolic Science, University of Cambridge, Box 285 Cambridge Biomedical Campus, Cambridge CB2 0QQ, UK.; ^3^ Genetics Department and Clinical Genomics Centre, Faculty of Medicine, Alexandria University, Alexandria, Egypt.; ^4^ Lilly Research Laboratories, Eli Lilly and Company, Indianapolis, IN 46225, USA.; ^5^ Precision Healthcare University Research Institute, Queen Mary University of London, London E1 1HH, UK.; ^6^ Section of Endocrinology and Investigative Medicine, Department of Metabolism, Digestion and Reproduction, Faculty of Medicine, Imperial College London, London W12 0NN, UK.

## Abstract

The glucose-dependent insulinotropic polypeptide receptor (GIPR) is a major therapeutic target in type 2 diabetes and obesity. Missense variation in *GIPR* could confer phenotypic effects through alterations to constitutive activity or functional responses to GIP or pharmacological agonists. In this study, we aimed to provide a deep understanding of the molecular mechanisms that underpin the cellular and physiological impacts of *GIPR* coding variation by studying 30 *GIPR* coding variants in cellular models and pancreatic islets. Many variants showed impaired GIP-induced cyclic adenosine monophosphate responses, and population-based association analysis highlighted that these loss-of-function variants decrease body mass index but increase glycemia. In many cases, reduced function was partly driven by reduced expression at the cell surface due to impaired stability and redirection toward proteasomal degradation. Molecular dynamics simulations suggest distinct variant-induced perturbations in inter- and intrahelical interactions, which interfere with receptor stability. This study highlights the mechanisms and consequences of *GIPR* coding variation, which may have implications for the therapeutic targeting of this receptor.

## INTRODUCTION

The glucose-dependent insulinotropic polypeptide receptor (GIPR) is a class B1 G protein–coupled receptor (GPCR) expressed particularly in pancreatic islets, adipose tissue, the brain, and several other tissues (*1*). Its native peptide hormone ligand, GIP(1-42), is released by intestinal K cells in response to nutrient ingestion, augmenting glucose-stimulated insulin secretion to regulate postprandial glycemia (*2*). Despite similar antihyperglycemic effects to the related gut hormone glucagon-like peptide-1 (GLP-1) and its pharmaceutical analogs, the GIP system has, until recently, attracted less attention as a therapeutic target in type 2 diabetes (T2D) and obesity (*3*). This stems partly from the observation that the insulinotropic effect of GIP is reduced or absent after the onset of T2D (*4*) but also from the still unclear role for GIPR in bodyweight regulation (*5*). Counterintuitively, both GIPR agonists and antagonists can promote weight loss in rodents (*6*, *7*), and these paradoxical findings have prompted the development of contrasting antiobesity strategies in which GLP-1R agonism is combined with either GIPR agonism (e.g., tirzepatide) or antagonism [e.g., maridebart cafraglutide (MariTide)] (*8*, *9*). The observed clinical efficacy of these agents seemingly vindicates these approaches, although how much they depend on GIPR action remains undetermined, particularly as there is a paucity of human data evaluating the effects of sustained pharmacological modulation of the GIP system in isolation.

Understanding of the physiological role of GIPR has been enhanced by genetic evidence accumulated over the past two decades. An early pivotal study demonstrated that *Gipr* knockout mice are resistant to weight gain when fed a high-fat diet (*10*), positioning GIPR antagonism as a potential therapeutic approach for weight loss. However, this effect is not seen when *Gipr* knockout mice are maintained at thermoneutrality (*11*). *Gipr* knockout mice show mild oral glucose intolerance, reflecting impaired β cell function (*12*). Three loss-of-function (LoF) human *GIPR* coding variants—Glu^354^→Gln (E354Q), Glu^288^→Gly (E288G) and Arg^190^→Gln (R190Q)—were previously reported to reduce body mass index (BMI) (*13*–*16*); E354Q is also associated with impaired glucose tolerance and reduced insulin secretion during an oral glucose tolerance test (*13*, *17*), but the effects of the E288G and R190Q variants on glycemia are less clear. Until recently, phenotypic and pharmacological effects of other *GIPR* coding variants were less well described, especially in comparison with *GLP1R* variants (*18*–*20*), but a recent report has provided a broader picture (*21*). The latter study showed that, with some caveats, LoF *GIPR* mutations typically lead to reductions in both BMI and adiposity but do not influence T2D risk.

Missense coding variation can affect GPCR function via several different mechanisms. The most common cause is reduced delivery of mutated receptors to the plasma membrane (*22*), and E288G, R190Q, E354Q, and many of the deleterious variants described by Kizilkaya *et al.* (*21*) show reduced surface expression (*15*, *16*, *21*, *23*), as do several *GLP1R* LoF variants (*18*–*20*). However, altered function may also result from a change in intrinsic ability to respond to GIP, as has been described for the E288G and R190Q *GIPR* variants (*15*, *16*). In contrast, E354Q shows an “overactivation” phenotype in which accelerated desensitization is thought to lead to LoF (*23*). Nuance in how variant GIPRs couple to G protein–dependent and G protein–independent pathways was recently highlighted as retained coupling to β-arrestin was able to avoid the phenotypic effects of some variants showing loss of cyclic adenosine monophosphate (cAMP) production (*21*). Some individual mutations can result in both mislocalization and intrinsic functional defects, so that disentangling the contribution of each can be challenging. Moreover, deeper molecular and structural understanding of why certain *GIPR* variants show reduced surface expression or altered function is lacking. In addition, except for E354Q (*24*–*26*), previous studies of *GIPR* coding variants have used human embryonic kidney (HEK) 293 and related expression systems that may not fully recapitulate how they behave in their native cellular environment.

In this study, we have aimed to address these unanswered questions by performing functional and genetic association analyses of 30 naturally occurring *GIPR* missense variants both in HEK293 and in INS-1 pancreatic β cells, accompanied by a study of selected variants in primary islets and molecular dynamics (MD) simulations to understand the structural basis of the observed effects.

## RESULTS

### *GIPR* missense mutations frequently lead to reduced expression at the cell surface

**Fig. 1.**
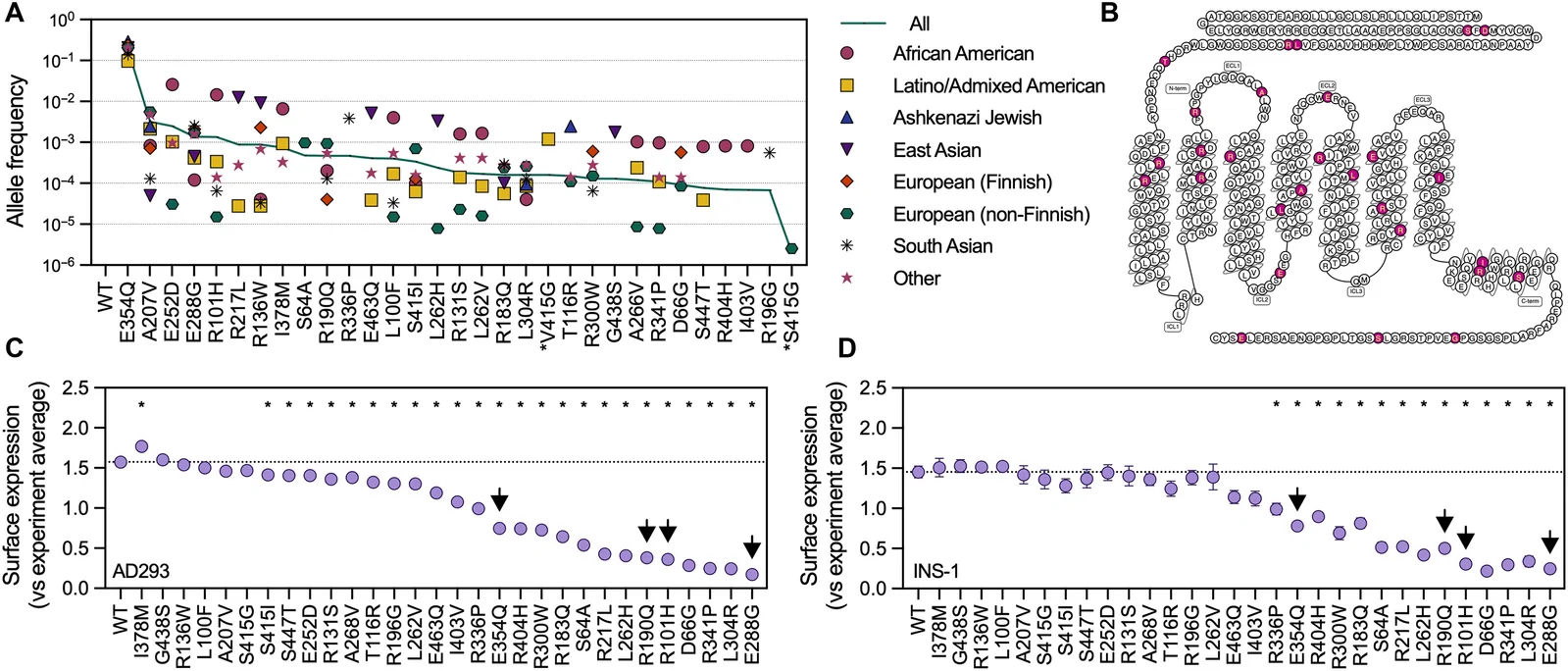
Surface expression levels of GIPR variants in different cellular models. (**A**) Allele frequency of selected *GIPR* variants in different genetic ancestral groups from gnomAD. Note that Val^415^→Gly (V415G) has a higher prevalence than Ser^415^→Gly (S415G), but as our “wild-type” (WT) receptor sequence uses serine at this position, prevalence data for S415G are also shown. (**B**) Positions of selected variants (from gpcrdb.org). (**C**) Mean surface expression of wild-type and variant SNAP-GIPR in AD293 cells, *n* = 41, with scaling to the experimental average and comparison by a one-way matched analysis of variance (ANOVA) with Dunnett’s test versus wild type. The dotted line shows the wild-type mean. Arrows highlight E354Q, R190Q, R101H, and E288G, which are investigated in more detail below. (**D**) As for (C) but in INS-1 cells, *n* = 13. *P < 0.05 using the indicated statistical test. All data represented as means ± SEM.

An initial selection of the most common 30 individual missense *GIPR* variants was made from gnomAD v2.1.1, a dataset that includes whole-exome sequencing (WES) and whole-genome sequencing (WGS) data from large-scale projects involving diverse populations (*27*). The allele frequency of the rarer variants diverged between different ancestral groups (Fig. 1A). Missense variants were distributed throughout the receptor’s extracellular, transmembrane, and intracellular domains (Fig. 1B).

To study the impact of these missense mutations on receptor surface expression, we obtained plasmids encoding wild-type and each variant GIPR with an N-terminal SNAP-tag to allow surface-specific fluorescent labeling of the receptor using cell-impermeable probes. We confirmed that the SNAP-tag did not alter the subcellular localization of GIPR in the basal state, binding of fluorescent GIP-TMR, or the cAMP accumulation response to GIP(1-42) (fig. S1, A and B). Note that, at position 415, major alleles encoding either serine (e.g., variant S415I) and valine (e.g., variant Val^415^→Gly (V415G)] are mapped (Fig. 1A); we used the serine residue as the “wild type” for functional studies, so henceforth refer to the glycine mutation as “S415G.” We first assessed variant cell surface expression in transiently transfected adherent HEK293 (AD293) cells using a cell-impermeant SNAP-tag probe to specifically label GIPR at the plasma membrane (fig. S1C). Here, 24/30 variants had a surface expression level that was statistically significantly below that of the wild-type receptor, with 13 of them showing <50% of the wild-type expression level (Fig. 1C). Surface expression of the same variants in INS-1 832/3 clonal β cells devoid of endogenous GIPR after CRISPR-Cas9 deletion (*28*) (referred to in this work as “INS-1 cells”) was strongly correlated with AD293 results (Fig. 1D and fig. S1D). Variants previously demonstrated to show reduced surface expression, including E354Q, R190Q, and E288G (*16*, *23*), performed as expected in our system. The second and third most common variants [Ala^207^→Val (A207V) and Glu^252^→Asp (E252D)] behaved similarly to wild-type GIPR, but the fifth most common (and the second most common in African American individuals in gnomAD), Arg^101^→His (R101H), showed markedly reduced expression.

We compared our results with those from Kizilkaya *et al.* (*21*). Of the 13 variants evaluated in both studies, 10 showed good agreement between surface expression measured by SNAP-tag labeling (our study) and *B*_max_ measured using radioligand binding for untagged GIPR [Kizilkaya *et al.* (*21*)] (fig. S1E). However, E354Q, A207V, and T116R showed divergent results. The discrepancy with E354Q is likely due to experimental variability in the radioligand binding assay as denoted by the reported wide SE bars (*21*). Additional experiments using SNAP-tagged GIPR reported by Kizilkaya *et al.* (*21*) also showed wild-type–like surface expression of the A207V and Thr^116^→Arg (T116R) variants, i.e., similar to our results. We therefore performed radioligand binding assays using a selection of untagged GIPR variants, including A207V, to rule out any variant-specific interference due to receptor tagging. In our hands, maximal binding for untagged A207V was similar to wild-type GIPR (table S1).

### Predicted thermal stability influences variant GIPR surface expression

**Fig. 2.**
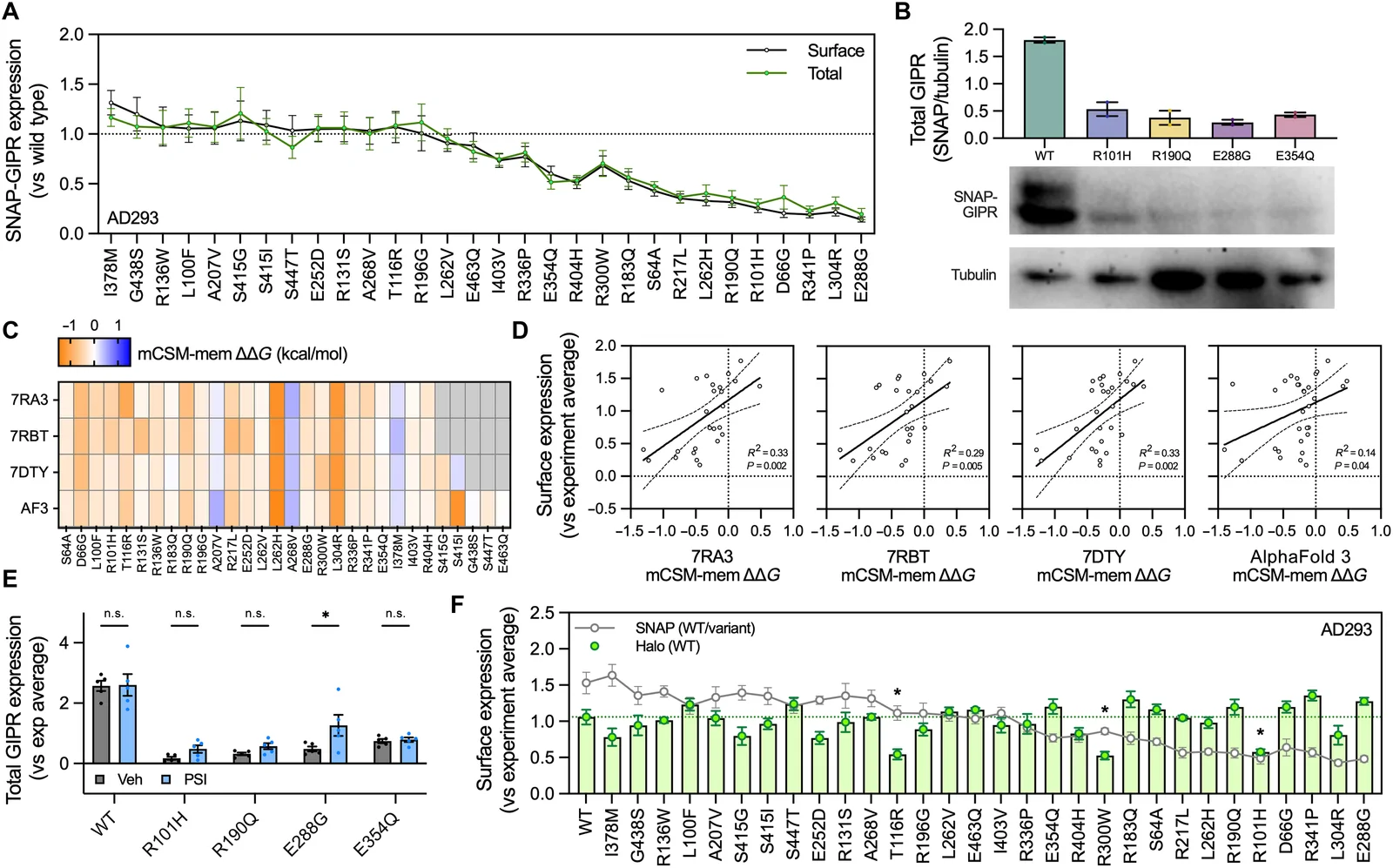
Investigations into mechanisms underpinning altered surface expression of GIPR variants. (**A**) Mean surface and total expression of wild-type and variant SNAP-GIPR in AD293 cells, *n* = 8, with normalization to the wild type (dotted line) and compared using a two-way matched ANOVA with Sidak’s test. (**B**) Western blot showing the total abundance of wild-type and variant SNAP-GIPR in INS-1 cells, with densitometric quantification of *n* = 2. (**C**) Heatmap showing the predicted effect on thermostability of each variant as assessed using the mCSM-membrane and the indicated experimental (7RA3, 7RBT, and 7DTY) and AlphaFold 3 (AF3) structures. Decreased stability is predicted when ∆∆*G* is negative, particularly <−0.5. (**D**) Association between the mCSM-membrane-predicted ∆∆*G* from (C) and the measured surface expression in AD293 cells (Fig. 1C). The linear regression line with 95% confidence intervals is shown. (**E**) Effect of proteasomal inhibition using PSI (100 μM, 4 hours) on total SNAP-GIPR levels in INS-1, *n* = 5, scaled to experimental average without PSI, and compared using a two-way matched ANOVA with Sidak’s test. (**F**) Mean surface expression of wild-type Halo-GIPR and wild-type/variant SNAP-GIPR in AD293 cells, *n* = 8, with scaling to the experimental average for each probe, statistical comparison of Halo-GIPR expression in the presence of variant compared to wild-type Halo-GIPR by a one-way matched ANOVA with Dunnett’s test. *P < 0.05 using the indicated statistical test. All data represented as means ± SEM. n.s., not significant.

Reduced surface expression of variant GIPR could result from several mechanisms, for example, misfolding during biosynthesis at the endoplasmic reticulum and subsequent degradation, changes to localization sequences, and accelerated turnover (*22*). To test for specific deficits in delivery of mature receptor to the cell surface, we labeled “total” SNAP-GIPR using a cell permeable probe, BG-OG (*29*), alongside standard surface-specific labeling. In AD293 cells, whole-cell SNAP-GIPR closely mirrored surface expression levels (Fig. 2A), with only small nonsignificant relative increases in total SNAP detected with the lowest-expressed variants. Moreover, total levels of SNAP-GIPR variants E354Q, E288G, R190Q, and R101H, selected for testing due to their relatively higher prevalence and genetically or experimentally confirmed phenotypic effects, were also significantly reduced, as shown by SNAP immunoblot of transfected INS-1 cells (Fig. 2B).

Reduced protein stability is major contributor to pathogenicity of human missense variants (*30*). Using three experimental GIPR structures (*31*, *32*) and the model predicted by AlphaFold 3 (*33*), we estimated the impact of each mutation on thermal stability using a tool optimized for transmembrane proteins (*34*). Using ∆∆*G* < −0.5 to indicate a destabilizing effect, three mutations [Asp^66^→Gly (D66G), Leu^262^→His (L262H), and Leu^304^→Arg (L304R)] were predicted to be destabilizing from all four structures and an additional eight from at least one structure (Fig. 2C). In each case, predicted effects on stability showed a significant association with surface expression, with better goodness of fit found with the experimental structures compared to the AlphaFold prediction (Fig. 2D). Of note, R190Q and R101H mutations produced ∆∆*G* < −0.5 with at least one structure, with E288G showing a borderline effect (mean ∆∆*G* = −0.366 from the experimental structures) and little change for E354Q. As unstable, misfolded nascent proteins in the biosynthetic pathway are frequently redirected from the endoplasmic reticulum toward proteasomal destruction (*35*), we assessed the effect of 4 hours of treatment with a proteasomal inhibitor, PSI (*36*), on whole-cell SNAP levels following transient transfection of SNAP-GIPR variants. Wild-type and E354Q GIPR were unaffected by proteasomal inhibition, but there was a significant increase in E288G levels, with numerically similar trends with R190Q and R101H (Fig. 2E). In contrast, inhibition of constitutive GIPR down-regulation using the lysosomal acidification inhibitor bafilomycin A1 (*37*) did not lead to disproportionate “rescue” for GIPR variants with reduced surface expression (fig. S2A). These data suggest that reduced plasma membrane levels of selected *GIPR* missense variants result primarily from effects on the biosynthesis or folding of mature GIPR proteins within the cell.

As most *GIPR* genetic variants are encountered in heterozygosity, we also tested for dominant negative effects by cotransfecting wild-type Halo-tagged GIPR with each SNAP-tagged variant and quantifying surface expression of both using spectrally distinct surface-specific probes (fig. S2B). Most variants had little effect on wild-type surface expression, but three [T116R, Arg^300^→Trp (R300W), and R101H] produced a significant reduction, suggesting dominant negative effects (Fig. 2F).

### Deciphering the role of *GIPR* variant expression defects on cAMP signaling

**Fig. 3.**
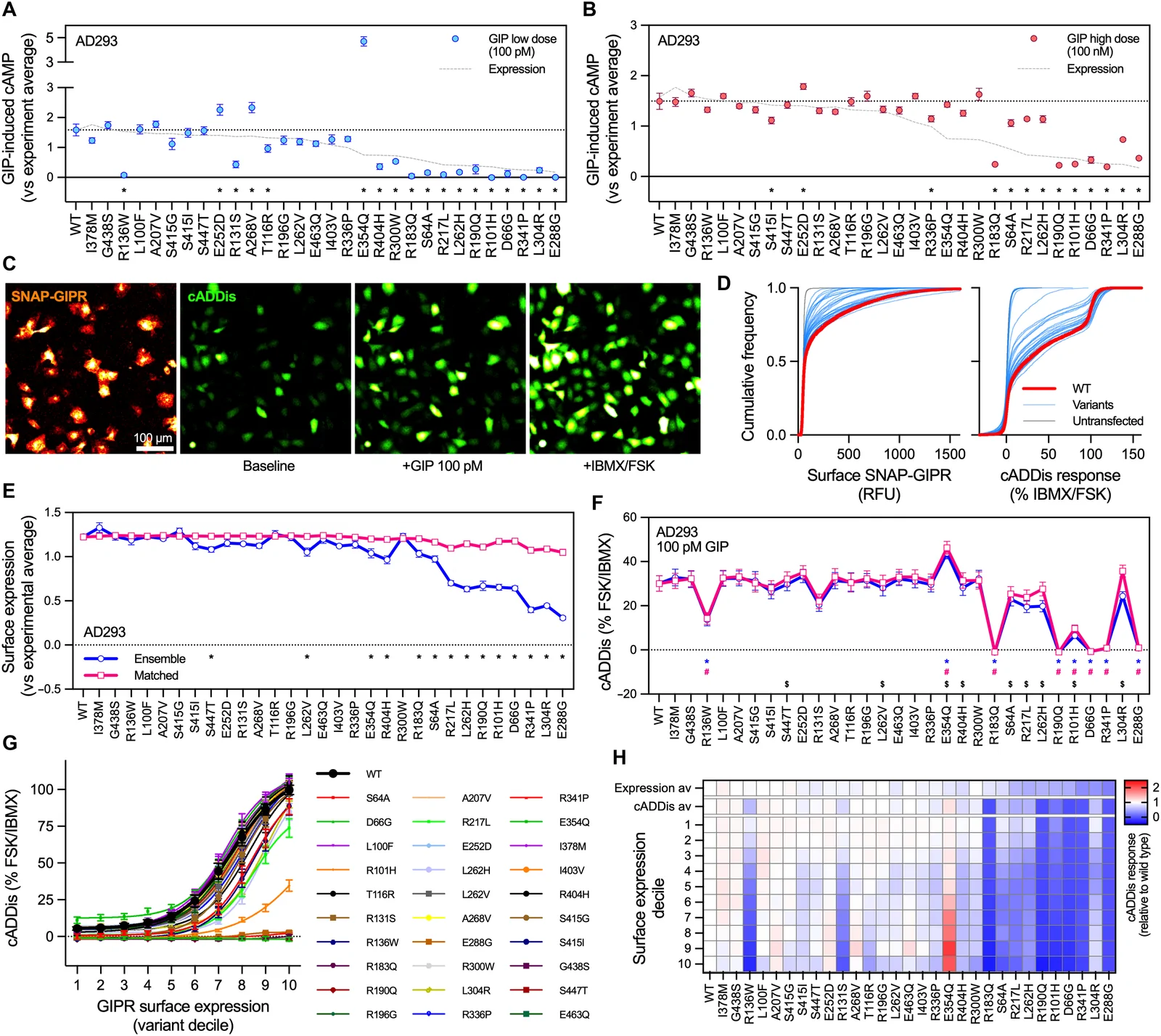
Rescue of cell surface expression does not fully restore cAMP signaling with all variants. (**A**) cAMP responses to 100 pM GIP, measured by HTRF in AD293 cells, *n* = 8. Results are scaled to the experimental average and compared by a two-way matched ANOVA with Dunnett’s test versus wild type. The dotted line shows the wild-type mean, and the dashed line shows the surface expression level from Fig. 1C. (**B**) As for (A) but 100 nM GIP. (**C**) Representative image showing cADDis imaging in SNAP-GIPR–transfected AD293 cells labeled with SNAP-Surface 647. Scale bar, 100 μm. (**D**) Cumulative frequency distributions showing surface GIPR labeling and cADDis responses from a single experiment. (**E**) Surface GIPR expression from ensemble (unselected) and expression-matched analysis of cADDis assay, *n* = 8, with scaling to experimental average and comparison by a two-way matched ANOVA with Dunnett’s test versus wild type. (**F**) cADDis response to 100 pM GIP from same experiments as (E), with a two-way matched ANOVA with Dunnett’s test versus wild type and Sidak’s test comparing ensemble and expression-matched data. (**G**) Alternative analysis data from (F) showing cADDis responses to 100 pM GIP stratified according to variant-specific surface expression deciles. (**H**) As for (G) but with normalization to the wild-type response at each variant-specific decile. *P < 0.05 (variant versus wild type from ensemble cells), ^#^*P* < 0.05 (variant versus wild type from expression-matched cells), and ^$^*P* < 0.05 (ensemble versus expression-matched cells), using the indicated statistical test. All data represented as means ± SEM.

As GIPR is canonically coupled to Gα_s_-mediated cAMP signaling, we next measured changes in cAMP by immunoassay in AD293 cells transfected with wild-type or variant GIPR in response to stimulation with 100 pM or 100 nM GIP, selected to represent physiological (*38*) and maximal concentrations, respectively. Expectedly, the 10 variants with the lowest surface expression showed reduced cAMP responses to both 100 pM and 100 nM GIP (Fig. 3, A and B). However, some of these [e.g., Ser^64^→Ala (S64A), Arg^217^→Leu (R217L), and L262H] showed at least a partially restored response at the higher ligand concentration. The cAMP response at 100 nM was closely associated with the ability of each variant to recruit mini-G_s_, a conformational sensor of GPCR activation (*39*) (fig. S3, A and B). Notably, Arg^136^→Trp (R136W) and Arg^131^→Ser (R131S) showed a reduced cAMP response to 100 pM GIP despite close to normal surface expression levels, suggestive of a primary functional deficit. In contrast, E354Q exhibited an increased response at 100 pM GIP despite reduced expression levels, in keeping with previous knowledge that this variant shows in vitro gain-of-function (GoF) characteristics despite displaying LoF-like physiological impacts (*23*). E252D and Ala^268^→Val (A268V) also showed GoF cAMP responses to 100 pM GIP. As the GIPR can in some cases have relatively high rate of constitutive activity (*40*), we measured cAMP from GIPR-expressing cells with addition of IBMX to boost basal cAMP accumulation without GIP ligand stimulation; however, there were no statistically significant differences found between variants and wild type in AD293 cells (fig. S3C). We also measured cAMP responses in INS-1 cells, which showed a similar pattern to that seen in AD293 cells (fig. S3D).

Although these data demonstrate a clear overall positive association between GIPR variant surface expression and cAMP response (fig. S3E), they provide an incomplete description of the deleterious impact of certain variants as impaired surface expression could disguise an additional functional deficit. Elucidating the relative contribution of each is potentially important as mutations that primarily affect trafficking to the plasma membrane could be amenable to rescue, e.g., by pharmacological chaperones (*41*). To resolve this question, we used the cell-to-cell heterogeneity in plasmid uptake during transient transfection to facilitate biosensor recordings of cAMP responses from individual cells with different levels of surface GIPR. This high-content imaging assay involves fluorescence imaging of thousands of cells in parallel after cotransduction of SNAP-tagged GIPR and the live cell cAMP sensor cADDis (*42*) (Fig. 3, C and D). cAMP responses to 100 pM GIP from “ensemble” (nonselected) AD293 cells measured in this assay agreed with the biochemical cAMP measurements, although the relationship was nonlinear due to the greater sensitivity of the Epac2-based biosensor (fig. S3F). We used a weighting procedure based on grouping variant-expressing cells into deciles matched to the surface expression of the wild type, allowing cAMP responses to be ascertained at equalized surface expression levels from different receptor variants (Fig. 3E). Notably, all variants with reduced ensemble cAMP responses still showed LoF after matching to wild-type surface expression levels, indicating that they all have intrinsic defects in cAMP generation (Fig. 3F). With others, however, responses were at least partly restored to wild-type levels, including R101H, S64A, R217L, L262H, R101H, and L304R. We interpret this as indicating that, for these variants, cAMP LoF reflects both reduced surface expression and intrinsically impaired GIP responsiveness. In contrast, other variants such as Arg^183^→Gln (R183Q), R190Q, D66G, and E288G showed no rescue of function even at wild-type–like expression density, even at a saturating concentration of GIP (100 nM), highlighting their profound functional defects (fig. S3, G and H).

Extending this approach further, we analyzed variant cADDis responses within their own deciles of surface expression, reasoning that this approach would give some insights into the possibility of cell- or tissue-specific variant activities. Absolute responses to 100 pM GIP are plotted per surface expression decile in Fig. 3G and reanalyzed in Fig. 3H to better show comparisons against the wild type. Some variants, especially E354Q (but to a lesser extent A207V, E252D, and A268V), showed increased responses relative to the wild type at their own lower bounds of expression. Others showed reduced responses relative to the wild type at lower expression levels but, at their own highest levels of expression, produced wild type–like responses, e.g., R136W, R131S, T116R, and S64A. Given the wide range of *GIPR* expression across different metabolic tissues, these data raise the possibility that GIPR variants could produce physiologically selective actions.

### Limited effects of missense variants on GIPR internalization

**Fig. 4.**
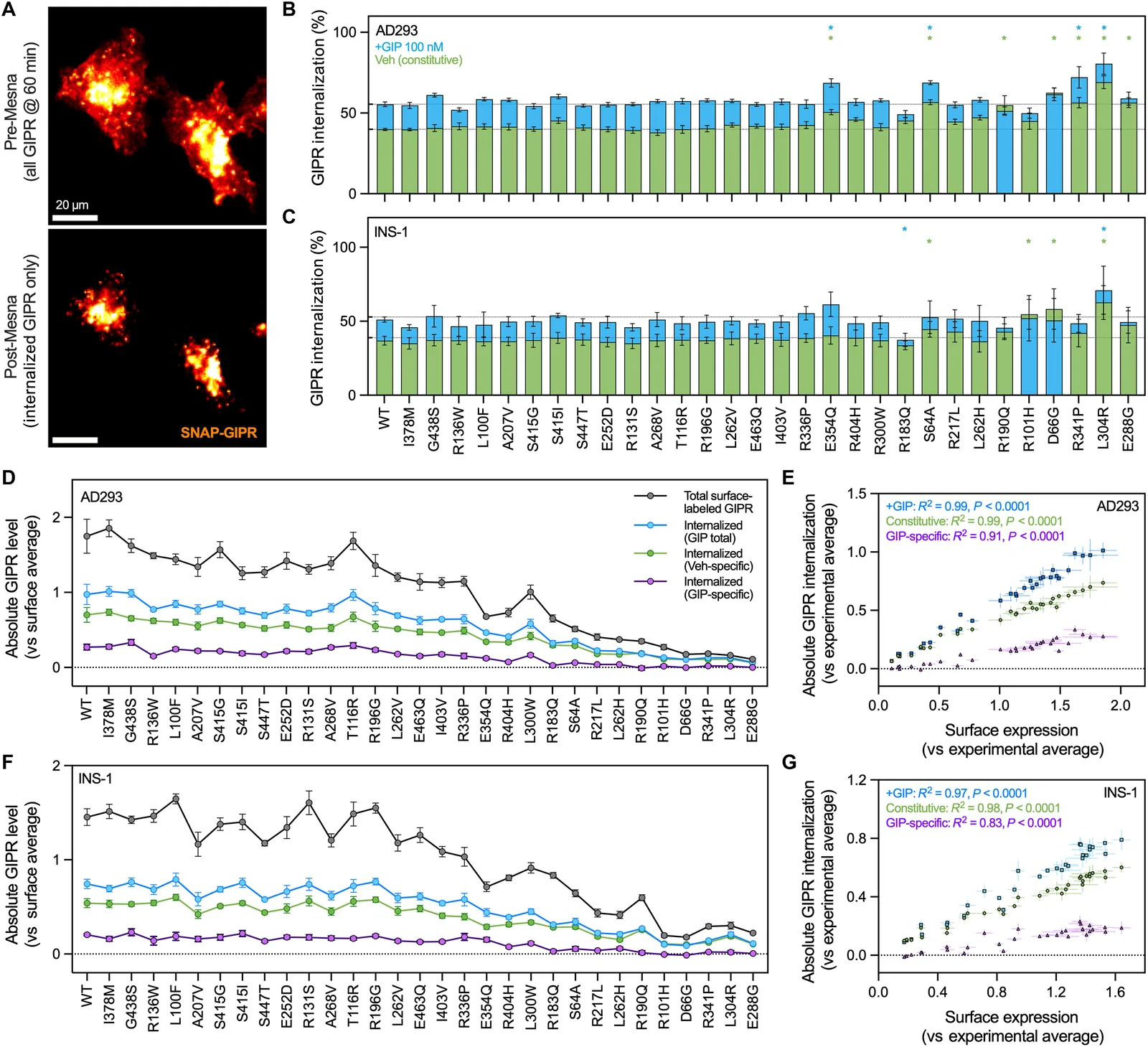
Constitutive and GIP-induced GIPR internalization effects. (**A**) AD293 cells expressing wild-type SNAP-GIPR, prelabeled with BG-SS-649, and treated for 60 min with 100 nM GIP; imaging performed before and after Mesna treatment. Scale bars, 20 μm. (**B**) Percentage of prelabeled SNAP-GIPR internalized over 60 min with vehicle or 100 nM GIP treatment in AD293 cells, *n* = 6, with comparison of variants versus wild type by a two-way matched ANOVA with Dunnett’s test. Dotted lines represent mean wild-type constitutive and GIP-stimulated responses. (**C**) As for (B) but in INS-1 cells, *n* = 7. (**D**) Quantification of the absolute amount of total and internalized surface-labeled GIPR in AD293 from the experiments shown in (B), highlighting the relative contributions of constitutive and GIP-specific internalization. Results have been scaled to the experimental average for total surface GIPR expression. (**E**) Relationship between the AD293 surface GIPR expression from Fig. 1A and the absolute amount of GIP-induced GIPR internalization amount from (D). (**F**) As for (D) but using INS-1 data from (C). (**G**) As for (E) but using INS-1 data from Fig. 1B and (E). *P < 0.05 by the indicated statistical test. All data represented as means ± SEM.

Preserved β-arrestin–mediated GIPR internalization has been suggested to influence *GIPR* missense variant phenotypic effects by driving endosomal signaling (*21*). We used a reversible SNAP-tag labeling technique (Fig. 4A) to quantify both constitutive and GIP-induced internalization of wild-type SNAP-GIPR in AD293 cells and INS-1 β cells. Of note, 40% of wild-type GIPR was constitutively internalized (i.e., without an agonist) over 60 min in AD293 cells, increasing to ∼60% in the presence of a maximal concentration of GIP (fig. S4A). Thus, an additional 20% of surface-labeled GIPR underwent internalization when the agonist was applied at a saturating concentration, with an EC_50_ (median effective concentration) almost 300 times higher than for cAMP production in this cell model (fig. S4B), in keeping with previous reports highlighting the relatively limited capacity for GIP-induced GIPR internalization (*43*, *44*). However, it should be noted that, despite this relatively small net increase in internalization, most of the receptor internalized at this saturating concentration will be bound to GIP.

Most *GIPR* missense variants showed wild type–like internalization in the presence of 100 nM GIP, with similar results with AD293 and INS-1 cell models (Fig. 4, B and C, and fig. S4D). Some moderate increases in internalization were recorded with selected low-expression variants, such as E354Q, S64A, Arg^341^→Pro (R341P), L304R, and E288G, which was driven by apparent increases in constitutive turnover. However, it should be emphasized that earlier experiments with bafilomycin A1 (fig. S2A) argue against accelerated constitutive down-regulation being a primary driver of reduced surface expression with these *GIPR* variants. When the GIP-induced increase in total internalization was analyzed separately, several variants including R190Q, R101H, E288G, D66G, and R183Q showed statistically significant reductions in internalization in either/both AD293 and INS-1 cells (fig. S4D). This provides further support for impaired intrinsic function of these variants, as suggested from the cAMP results in Fig. 3F.

Although percentage of internalization is helpful to understand functional deficits, the absolute amount of internalized GIPR may also be informative. In our analysis, it was both clear and expected that the amount of GIPR internalization (constitutive and GIP-mediated) was strongly dependent on variant-specific surface expression levels (Fig. 4, D to G). As capacity for endosomal signaling is, by definition, influenced by the total bulk of internalized agonist/receptor complexes, differences in surface expression are likely to play a major role in controlling the relative ability of *GIPR* variants to generate signals from intracellular compartments.

### MD simulations provide insights into structural basis for variant effects

Having demonstrated that several GIPR variants show both impaired surface expression (perhaps due to thermodynamic instability) and compromised ability to respond to GIP, we aimed to understand the possible structural basis for these effects. To this end, we performed microsecond-scale MD simulations based on a wild-type GIP-bound structure and four mutants, prioritized because of their prevalence and phenotypic effects (R190Q, E354Q, E288G, and R101H) to assess a potential structural impact at the molecular level.

**Fig. 5.**
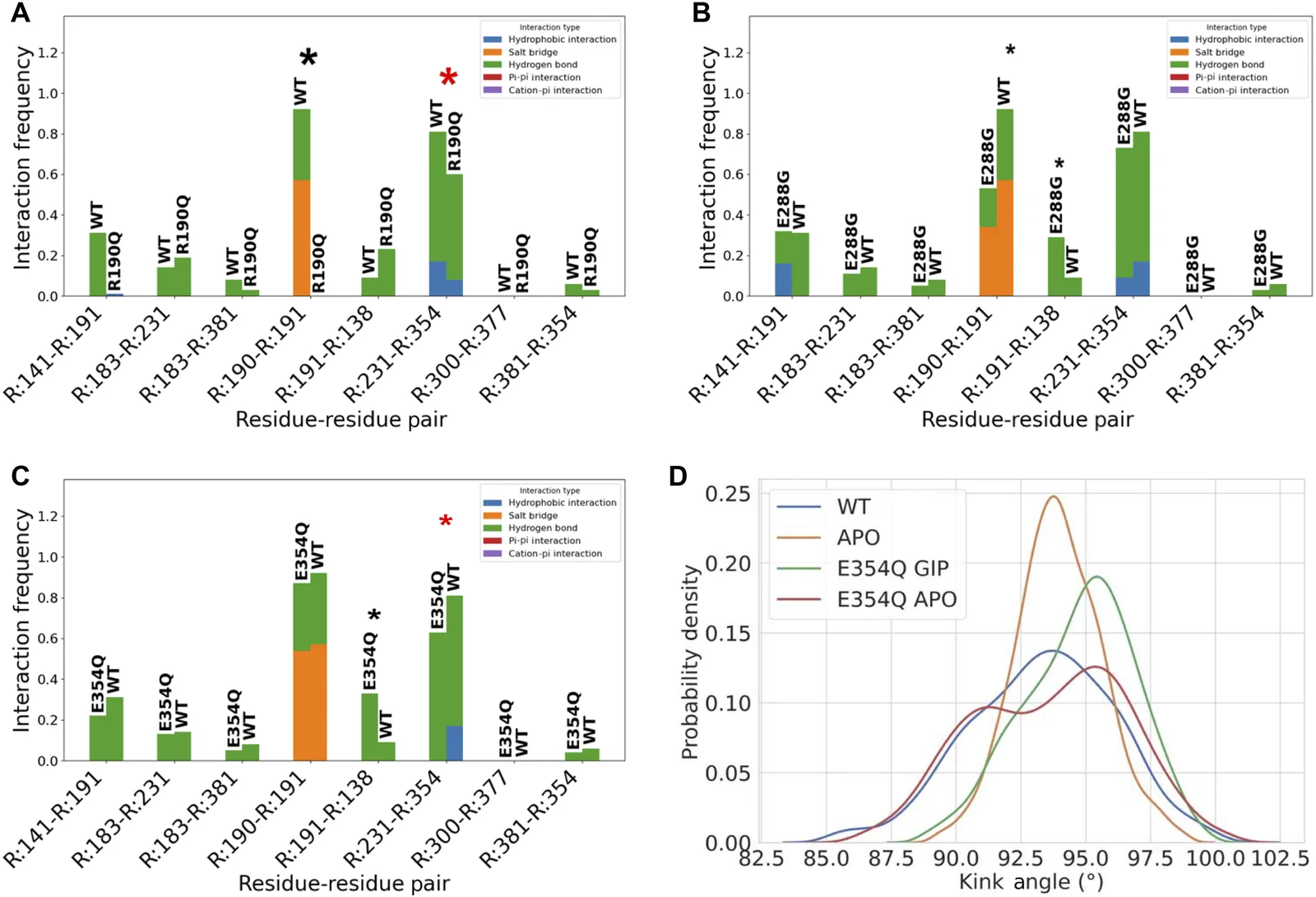
Protein-protein interactions from MD simulations. (**A**) Mutation R190Q disrupts both local and distant interhelical interactions within the GIP binding site. The local intrahelical interaction between R190 and D191 (indicated on the figure as R:190-R:191, with “R:” here referring to “receptor”) is completely lost in the presence of the Q190 mutant. This change also disrupts the TM1:Y141–TM2:D191 interhelical interaction (“TM1” refers to transmembrane helix 1) observed in the wild-type receptor. The interhelical interaction between TM4:Y231 and TM6:E354 is also moderately disrupted suggesting long-range effects of this mutation. (**B**) Mutation E288G produces intra- and interhelical interactions like that of the wild-type APO receptor (see fig. S5A). The most noteworthy change in residue-residue interactions comes from the central R190-D191 interaction on TM2. Microsecond simulations show a movement of the GIP peptide across the receptor to reduce the pi-cation interactions of GIP:F6 and GIP:I7; as well as engage in a new hydrogen bond between the backbone carbonyl of E288G and GIP:S8 side-chain hydroxyl (see fig. S5B). (**C**) Mutation E354Q shows moderate disruption of wild-type protein-protein interactions. The TM4:Y231–TM6:E354 interaction is key to stabilizing the GIPR bundle in the presence of GIP shown from the high-frequency hydrogen bonding and hydrophobic interactions between residues in the wild-type simulations. Introducing the E354Q mutation reduces but does not destroy this interhelical interaction. Long-range effects are also minimal with a slight enhancement in the TM1:Q138–TM2:D191 interaction. (**D**) TM6 kink angle defined by the vectors from residues 343 to 348 and 348 to 353. The shift in probability from 93.7° (more kinked) to 95.6° (less kinked) for the wild-type and E354Q variant, respectively, indicate a decrease in probability of finding the receptor in the active state in the presence of native peptide.

Although none of these variants make direct hydrogen bonding interactions with the GIP peptide, we chose several residue-residue interaction frequencies as observables to monitor the interhelical stability of the transmembrane peptide binding domain over simulation time. R190Q disrupts both local and distant interhelical interactions within the GIP binding site. The local intrahelical interaction between wild-type R190 and D191 is completely lost in the presence of this variant (Fig. 5A). This change also disrupts the TM1:Y141–TM2:D191 interhelical interaction observed in the wild-type receptor. In addition, the interhelical interaction between TM4:Y231 and TM6:E354 is moderately disrupted, suggesting long-range effects of this mutation. These long-range disruptions suggest that not only the size of the residue in position 190 is important but also the change and are consistent with experimentally measured reductions in surface expression and cAMP production. E288G produces intra- and interhelical interactions like that of the wild-type APO (ligand-free) receptor (Fig. 5B and fig. S5A). The most noteworthy change in residue-residue interactions comes from the central R190-D191 interaction on TM2. Microsecond simulations show a movement of the GIP peptide across the receptor that reduces the pi-cation interactions of GIP:F6 and GIP:I7, as well as engage in a new hydrogen bond between the backbone carbonyl of E288G and GIP:S8 side-chain hydroxyl (fig. S5B). This result is consistent with observed variant expression defects on cAMP signaling, noting E288G’s only partly rescued function even at 100 nM GIP. E354Q shows moderate disruption of protein-protein interactions (Fig. 5C). The TM4:Y231–TM6:E354 interaction is key to stabilizing the GIPR bundle in the presence of GIP, shown from the high-frequency hydrogen bonding and hydrophobic interactions between residues in the wild-type simulations. Introducing the E354Q mutation reduces but does not destroy this interhelical interaction. Long-range effects are also minimal with a slight enhancement in the TM1:Q138–TM2:D191 interaction. R101, located in the extracellular domain, shows minor disruption of long-range protein-protein interactions at TM4-TM6 in the R101H variant form (fig. S5C). Although R101H does not disrupt the local orthosteric interactions typically observed in the presence of GIP, there is a small consistent long-range effect. All the variant simulation data suggest that the TM6 E354–TM4:Y231 interhelical bundle interaction is sensitive to changes in receptor sequence. Because E354 is located on TM6, directly implicated in signal transmission through the TM6 kink, even long distance (both sequentially and spatially) could influence G protein engagement.

To understand the role that the TM6:E354–TM4:Y231 interhelical interaction, the E354Q variant, and modulators thereof play in protein stability and dynamics, we used the angle defined by the vectors from residues 343 to 348 and 348 to 353 to represent the TM6 “kink.” A probability distribution was calculated over simulation time as a function of this angle for both the wild-type and the E354Q variant (Fig. 5D). The shift in peak probability from 93.7° (active reference) to 95.6° (less kinked, less active) for the wild-type and E354Q variant, respectively, suggests lower probability of finding the receptor in the active state in the presence of native peptide. The APO E354Q variant exhibits a modified probability distribution compared to the wild-type APO receptor resembling that of a GIP bound wild-type receptor. The bimodal distribution is characteristic of a more dynamic system and points toward a potential increase in constitutive activity, as hinted at by moderately increased basal turnover of this variant in our internalization assays (Fig. 4).

### Assessment of *GIPR* variant function in pancreatic islets

**Fig. 6.**
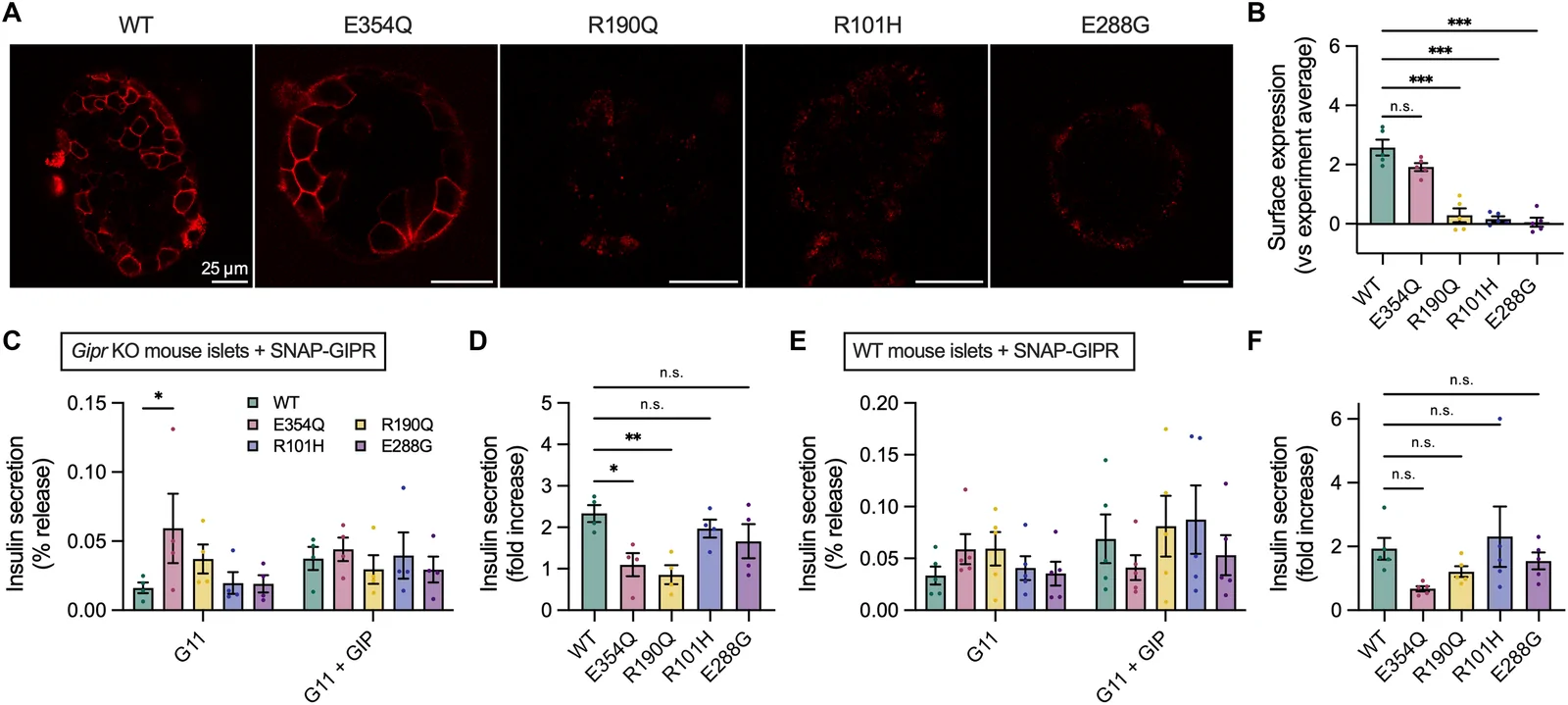
Evaluating variant effects in pancreatic islets. (**A**) Representative confocal images showing the surface labeling of wild-type and variant SNAP-GIPR in mouse pancreatic islets after adenoviral transduction. (**B**) Quantification of surface SNAP-GIPR from *n* = 5 repeats and comparison by a one-way matched ANOVA with Dunnett’s test versus wild type. (**C**) Insulin release as a percentage of total insulin content from *Gipr*^−/−^ mouse islets transduced with GIPR adenoviruses at 11 mM glucose (G11), with and without 100 nM GIP, 30-min stimulation, *n* = 4. Wild-type and variant responses compared by a two-way matched ANOVA with Dunnett’s test. (**D**) Data from (C) analyzed as GIP-induced fold change, with comparison using a one-way matched ANOVA with Dunnett’s test. (**E**) As for (C) but in wild-type mouse islets after adenoviral transduction, *n* = 5. (**F**) As for (D) but analyzing insulin data from (E). *P < 0.05; **P < 0.01; ***P < 0.0001, using the indicated statistical test. All data represented as means ± SEM with individual data points.

To build on our functional screening results from AD293 and INS-1 cells, we examined the effects of selected variants in a primary pancreatic islet environment. Focusing on higher-prevalence variants with altered function in cellular models, we confirmed the expected surface expression patterns of E354Q, R190Q, R101H, and E288G using adenoviral SNAP-GIPR expression in mouse islets, with marked reductions seen particularly with the latter three variants (Fig. 6, A and B). We noted that both α and β cells were successfully transduced (fig. S6A), concordant with the endogenous expression pattern of GIPR, which directly regulates release of insulin and glucagon (*45*). Therefore, we performed insulin and glucagon release assays in islets from *Gipr* knockout mice transduced with wild-type or variant SNAP-GIPR to examine secretory effects in the absence of the endogenous receptor. We found that E354Q expression led to a significant increase in constitutive insulin secretion at 11 mM glucose (Fig. 6C), compatible with a higher constitutive activity level of this variant as hinted at by the internalization assays shown in Fig. 4 and the MD results in Fig. 5D. However, E354Q showed a blunted insulinotropic response to GIP stimulation compared to wild-type GIPR (Fig. 6D), in keeping with the reduced C-peptide response to oral glucose reported in people carrying this variant (*17*). R190Q, R101H, and E288G showed apparent reduced GIP-induced insulin secretion, although this was statistically significant only for R190Q. Each variant also showed apparent but nonstatistically significant reduction in glucagon release at 0.5 mM glucose compared to the wild type (fig. S6, B and C). We also assessed responses in islets from wild-type mice, in which the mouse GIPR is expressed endogenously, with the aim of modeling variant effects when expressed in heterozygosity with the wild-type receptor (albeit mouse in this case) (Fig. 6, E and F). Although not statistically significant, the same trends were observed, with a tendency toward reduced responses to GIP stimulation particularly with E354Q and R190Q. Binding of the fluorescent GIP analog GIP-TMR (*44*) was also tested in wild-type islets transduced with SNAP-GIPR adenovirus, showing high levels of binding to the wild-type and the E354Q variant but very low levels with the other variants, which were numerically reduced compared to even nontransduced control, hinting at possible dominant negative effects when overexpressed (fig. S6, D and E).

### Population-based validation of *GIPR* variant effects

To assess the phenotypic impact of the 30 *GIPR* missense variants, we turned to UK Biobank (UKBB), in which 28 of our 30 functionally characterized variants were represented. We performed rare variant burden testing using STAAR (*46*) to identify potential associations with BMI, glycated hemoglobin levels (HbA1c, in individuals without T2D), and T2D of distinct *GIPR* variant groups classified according to our in vitro data, specifically the 100 pM GIP cAMP responses and levels of surface expression in AD293 cells. We assigned variants to reduced, neutral, and increased response groups, with “reduced” defined as a statistically significant reduction of at least 50% below that of the wild-type receptor and “increased” as a significant increase above the wild type. BMI was included as a covariate to determine associations with T2D and HbA1c independently of BMI. The common variant E354Q was excluded from burden tests due to its high frequency. All other individual variants had a minor allele frequency (MAF) of <0.2% in the full multiethnic UKBB sample. Results on Europeans-only and full multiethnic UKBB samples were consistent, and statistical power was enhanced in the latter by its larger sample size and inclusion of rare variants that are more prevalent in non-Europeans. Results were also consistent in generalized linear regression sensitivity models. The following results are from STAAR models in the full multiethnic UKBB sample. Results from all burden testing analysis models are presented in table S2.

**Fig. 7.**
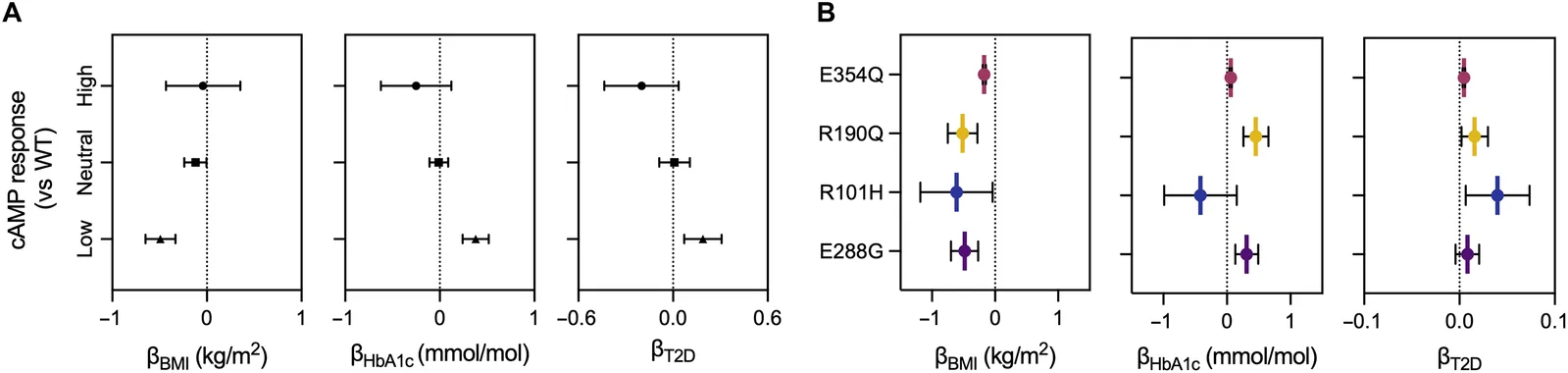
Contrasting effects of GIPR missense variants on BMI and glycemic status. (**A**) Results of the burden test analysis, showing the effects of *GIPR* variants grouped by cAMP response category on BMI, HbA1c (BMI-adjusted), and T2D risk (BMI-adjusted). (**B**) Results of selected single-variant association analyses, showing the effects of E354Q, R190Q, R101H, and E288G on BMI, HbA1c (BMI-adjusted), and T2D risk (BMI-adjusted). Data are represented as means ± 95% confidence intervals.

As summarized in Fig. 7A, rare *GIPR* variants that conferred reduced cAMP signaling (14 variants, up to 3867 carriers) were collectively associated with lower BMI (*P* = 8.4 × 10^−10^) but higher BMI-adjusted HbA1c (*P* = 3.9 × 10^−8^), with a nominal association with higher BMI-adjusted T2D risk [odds ratio (OR) = 1.21, *P* = 4.1 × 10^−3^]. By contrast, rare *GIPR* variants with neutral effects on cAMP signaling (11 variants, up to 7047 carriers) showed only nominal collective association with lower BMI (*P* = 2.8 × 10^−2^) and no association with HbA1c (*P* = 7.4 × 10^−1^) (Fig. 7A). No collective association was found with the two rare variants that conferred increased cAMP responses (E252D and A268V), although a directionally consistent trend toward reduced HbA1c and T2D risk was seen. The associations of *GIPR* in vitro LoF with increased HbA1c and T2D risk were consistent with or without adjustment for BMI (fig. S7A). We made similar observations when categorizing variants according to surface expression, with lower surface expression variants (11 variants, up to 3656 carriers) associated with reduced BMI (*P* = 7.0 × 10^−11^) and increased risks of BMI-adjusted HbA1c (*P* = 3.6 × 10^−7^) and BMI-adjusted T2D (*P* = 3.5 × 10^−3^) (table S2).

To assess individual variant impacts on the same outcomes, we performed single-variant association testing in the full multiethnic UKBB WES sample using BOLT-LMM v2.4.1 (*47*), with BMI included as a covariate for T2D and HbA1c associations, as above. Results from the four most common LoF variants (E354Q, R190Q, R101H, and E288G) are shown in Fig. 7B, with full data in table S3. In keeping with a previous report (*13*), the common E354Q variant (MAF = 19%, up to 178,179 carriers) showed statistically robust but relatively modest effects on decreased BMI (*P* = 3 × 10^−49^) and increased BMI-adjusted HbA1c (*P* = 3.2 × 10^−9^) and T2D risk (*P* = 8 × 10^−12^), directionally consistent with the above LoF variant groups. E288G (MAF = 0.18%, up to 1715 carriers) and R190Q (MAF = 0.15%, up to 1373 carriers) also showed significant effects on decreased BMI (*P* = 6 × 10^−6^ and *P* = 1.6 × 10^−5^, respectively) and increased BMI-adjusted HbA1c (*P* = 6.9 × 10^−4^ and *P* = 9.4 × 10^−6^, respectively) but with larger effects than E354Q, indicative of more marked impacts on receptor function. Even without BMI adjustment, R190Q and E288G led to increases in HbA1c (fig. S7B). The rarer R101H variant (MAF = 0.02%, up to 227 carriers) showed a nominally significant association with lower BMI (*P* = 0.036) but not with HbA1c (*P* = 0.15).

Overall, these data highlight that LoF *GIPR* variants decrease body weight but increase glycemia, and the latter effect is more pronounced after accounting for changes in BMI. Furthermore, despite conferring increased cAMP response to GIP, the common E354Q variant shows phenotypic consequences similar to the other LoF variants. A summary of the surface expression, pharmacology, islet effects and human phenotypic data for the key variants is provided in table S4.

### Similar impacts of GIP and tirzepatide on *GIPR* variant signaling responses

**Fig. 8.**
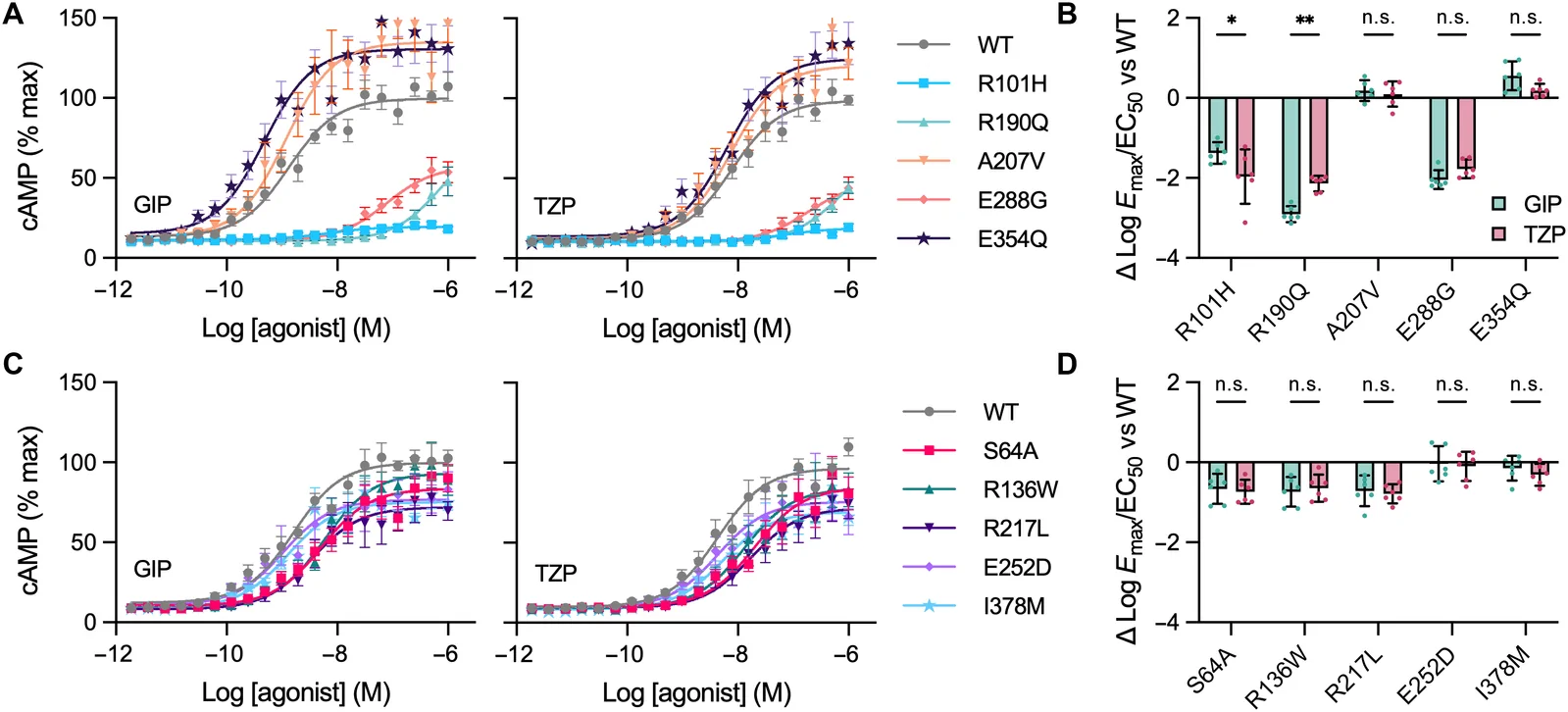
cAMP signaling with tirzepatide. (**A**) cAMP responses in AD293 cells expressing wild-type or variant SNAP-GIPR in response to GIP(1-42) or tirzepatide (TZP), *n* = 6. Assays were performed in the presence of 0.1% BSA. (**B**) Variant effect on cAMP signaling analyzed using the ∆Log*E*_max_/EC_50_ method, with statistical comparison of GIP versus tirzepatide effect by a two-way matched ANOVA with Sidak’s test. (**C**) Same as (A) but different selection of variants. (**D**) Same as (B) but different variants. *P < 0.05; **P < 0.0001, using the indicated statistical test. Concentration-response data are represented as means ± SEM, whereas ∆Log*E*_max_/EC_50_ data are shown as mean ± 95% confidence intervals to highlight differences for variant compared to wild type; individual data points are shown in some cases.

GIPR agonism is an important component of tirzepatide action (*48*, *49*), but to our knowledge, little is known about the impact of *GIPR* variation on tirzepatide responses, either using pharmacological assays or in the context of a clinical trial. Focusing on the 10 highest-prevalence variants from gnomAD, we therefore tested cAMP concentration responses to GIP or tirzepatide stimulation in AD293 cells (Fig. 8, A to D). Using the ∆Log*E*_max_/EC_50_ method to quantify variant effects (*50*), E354Q showed increased cAMP signaling, whereas E288G, R190Q, and R101H showed markedly reduced responses to both ligands (Fig. 8B). We observed statistically significant differences between GIP and tirzepatide with the R101H and R190Q variants, the importance of which is unclear as both ligands were very weak agonists in both cases. S64A, R136W, and R217L responses were moderately reduced compared to wild-type GIPR for both GIP and tirzepatide (Fig. 8, C and D). We confirmed selected variant cAMP signaling effects using untagged GIPRs (table S5), finding similar relative potency shifts to those seen with SNAP-tagged GIPR variants in Fig. 8.

Thus, across the tested *GIPR* variants, the functional disruptions in cAMP responses to tirzepatide appear to be similar to those in response to GIP(1-42).

## DISCUSSION

Here, we have performed a detailed characterization of 30 *GIPR* missense variants, including pharmacological, cell biological, structural, and genomic effects in both AD293 and INS-1 β cells. We found that a substantial proportion of missense mutations led to significant changes in cAMP signaling, driven jointly by impaired surface delivery of mature receptor to the plasma membrane and intrinsic functional defects. Our structural analysis supports a model in which multiple GIPR variants destabilize the active conformation through disruption of a conserved TM6-TM4 network, providing mechanistic insight into how distal mutations compromise GIP signaling. Leveraging human biobank genetic data allowed us to establish phenotypic correlates of altered GIPR function, which confirmed the reported effect of *GIPR* LoF to lower body weight but also robustly identified effects of *GIPR* LoF on increased glycemia and T2D risk.

Interest in understanding the effect of coding mutations on GPCR function has increased in recent years, aided by the wide availability of exome sequencing data. Important information has been gleaned from coding variants predicted to be highly deleterious, e.g., due to frameshifts, splice site mutations, or premature truncations, as such mutations are very likely to produce a null receptor phenotype (*15*). However, such mutations are usually very rare. In contrast, more subtle missense variants account for a larger proportion of genomic variation, but their effects are often not straightforward to predict, even with the generation of AI-based tools such as AlphaMissense (*51*). Experimental studies are often required to classify variants to delineate their phenotypic effects by combining functionally similar variants into groups to increase statistical power. Our functional characterizations led to far clearer phenotypic associations than those achieved by in silico bioinformatic predictions, for example, compared to reported minimum *P* values for predicted *GIPR* masks for BMI (*P* = 7.0 × 10^−4^) and HbA1c (*P* = 3.5 × 10^−3^) in the same UKBB population (500k WGS v2) (*52*).

Missense variants in the class B1 GPCR family have attracted recent attention, with a series of recent studies focused on *GLP1R*, *GCGR*, and *GIPR* (*19*–*21*, *53*, *54*). Each of these studies have highlighted altered surface expression of missense variants as a key driver to altered function. In the present work, we confirmed that many *GIPR* missense variants fail to express at adequate levels at the plasma membrane, and we provide insights into the reasons for these changes. In line with a recent study (*30*) evaluating a wide range of genes, our results point to reduced protein stability leading to degradation during biosynthesis, rather than defects associated with mistargeting to different intracellular locations or accelerated constitutive internalization/turnover leading to increased lysosomal degradation as a major underlying driver for the reduced levels of missense GIPR variants at the plasma membrane. A prominent role for β-arrestin–driven endosomal signaling in mediating the effects of *GIPR* variants was recently reported (*21*), which has not been examined in our study. We did, however, evaluate GIPR internalization, for which the agonist-dependent fraction is thought to be at least in part β-arrestin dependent (*55*, *56*). The wild-type GIPR shows a high constitutive internalization rate, which is only moderately increased in response to agonist, and with EC_50_ for steady-state internalization (which measures receptors retained intracellularly after 1 hour of vehicle or agonist exposure but does not account for individual receptors cycling in and out of the endocytic compartment) measured in the tens of nanomolar range, it is challenging to study these processes at physiologically relevant GIP concentrations in the picomolar range. It is plausible that, in addition to the small degree of GIP-induced potentiation of intracellular GIPR localization, a proportion of GIP/GIPR complexes might also enter the endocytic pathway via constitutive receptor internalization, without requiring β-arrestins, but still being available to contribute to signaling from intracellular compartments. Because our internalization assays directly read out the amount of internalized receptor, as well as the percentage, we could easily observe how large between-variant differences in surface expression are a major limiting factor to the achievable total amount of GIPR that can be delivered to endosomes. Further work is needed to understand the precise nature and downstream impacts of both wild-type and variant GIPR signaling at diverse intracellular compartments, as well as effects of acute over prolonged periods of vehicle, GIP, or long-acting GIPR agonist stimulation.

Our integrative simulation analysis provides a structural framework for understanding how naturally occurring GIPR variants contribute to altered receptor function. Although the prioritized mutations do not directly contact the GIP peptide, each induces distinct perturbations in inter- and intrahelical interactions within the transmembrane region, with consequences for receptor stability and signaling. Notably, the R190Q variant disrupts local TM2 interactions and long-range stabilizing contacts with TM1 and TM6, correlating with reduced surface expression and signaling output. Similarly, E288G, although structurally reminiscent of the wild-type apo state, perturbs critical intra-TM2 interactions and alters peptide positioning, consistent with its partial functional rescue. The E354Q variant impairs a key TM6-TM4 interhelical contact, leading to a subtle yet functionally relevant shift in the TM6 kink angle distribution toward a less active conformation. This effect on receptor dynamics highlights the sensitivity of the TM6-TM4 interface in maintaining active-state geometry. Last, the modest but reproducible long-range perturbations observed in the extracellular R101H variant suggest that surface-proximal residues can allosterically modulate transmembrane packing.

In the context of substantial interest in GIPR antagonism as a therapeutic approach for obesity, our results from LoF *GIPR* variants may shed light on potential metabolic effects of this strategy as both LoF variants and antagonists act by suppressing the action of the native GIP hormone. Although the weight-lowering effects of LoF *GIPR* variants are now established (*14*–*16*, *21*), their glycemic effects have been less clear. Although the common E354Q variant was already known to confer mild impairments in glucose tolerance (*13*), the unusual receptor pharmacology of this variant has limited its confident categorization as LoF. Unlike other LoF variants, we found that E354Q confers increased cAMP response to GIP and increased constitutive insulin secretion. However, by grouping functionally similar variants, we find that E354Q confers robust phenotypic associations with BMI, HbA1c, and T2D, similar to the other *GIPR* LoF variants. Our observed effects of *GIPR* LoF on increased HbA1c and T2D risk contrast with the expected glucose-lowering effects of reduced body weight. One explanation for these paradoxical observations is that reduced β cell GIPR activity leads to impaired insulin secretion. Alternatively, with increasing evidence that GIPR plays a role in glucose uptake by adipocytes (*48*, *57*, *58*), GIPR LoF might impair insulin sensitivity or some other aspect of adipocyte biology to modulate whole body glucose homeostasis.

The success of tirzepatide, a dual GLP-1R/GIPR agonist, has led to great interest in the possibility that GIPR agonism may also be effective for weight loss. Although much of the weight loss efficacy of tirzepatide may be due to G protein–biased agonism at the GLP-1R (*59*), several rodent studies have demonstrated the potential for weight loss with GIPR mono-agonism (*7*, *60*, *61*), and recent works show that long-acting GIPR agonists can drive a moderate degree of weight loss in humans (*62*, *63*). The latter study was notable in that weight loss seen with the long-acting GIPR agonist LY3537021 appeared to be via an anorectic mechanism but without the nausea typically seen with GLP-1R agonists. There is limited understanding as to how weight loss results from both GIPR agonists and antagonists, with one possibility being that sustained GIPR agonist exposure results in an antagonist-like effect via pronounced GIPR desensitization (*64*). On the other hand, it has also been suggested that different central nervous system neuronal populations are involved in driving the weight loss effects of GIPR agonists versus antagonists (*65*). Although LoF mutations are a useful proxy for predicting GIPR antagonist effects in humans, we believe that GoF variants are less useful as a proxy for GIPR agonism, mainly due to the quite different half-lives between native GIP and long-acting pharmacological agonists. Intermittent, postprandial exposure to native GIP followed by periods of resensitization is unlikely to replicate the effects of continuous pharmacological agonist exposure (*57*). There remains a need for more studies using pharmacologically optimized GIP-specific ligands rather than GLP-1/GIP combinations to provide a clearer picture of the overall effects on body weight control and glucose homeostasis, including evaluation of the potential effects of biased ligands that maintain cAMP signaling while reducing β-arrestin and internalization responses (*66*).

There are a number of limitations to our work. First, although our concordant results from HEK293 and INS-1 cells provide some confidence that GIPR variant behaviors translate between cell types, exogenous expression cannot capture the nuances of how the receptor is subject to transcriptional and functional regulation when expressed endogenously. Inherent differences in GIPR expression between cell types is one possibility, with variants showing reduced inherent signaling efficacy likely to produce disproportionately weak responses in tissues with low receptor reserve (*67*); we provide some estimation of these effects in Fig. 3. However, many other factors such as coexpression of other membrane proteins such as RAMPs, membrane lipid composition, and availability of downstream effectors inevitably contribute (*68*). Related to this, a further limitation is that much of the data presented relates to proximal receptor behaviors such as signaling and trafficking. Although we have assessed effects of selected variants on islet hormone release in Fig. 6, understanding how variants influence physiologically important downstream functions in other tissues will require additional work. To study GIPR variant behaviors in their native environment, CRISPR-Cas9 editing of the endogenous *GIPR* sequence in relevant cellular models, or ideally in a whole animal model, is needed. Targeted mutation knock-in mouse models are frequently increasingly used for this purpose, including for GIPR and GLP-1R (*26*, *69*), although species-specific differences, especially for GIPR (*70*), mean that this approach is not foolproof. Ultimately, experimental medicine studies stratified by genotype, and pharmacogenomic analysis of large clinical trials featuring GIPR agonists or antagonists, are needed to complement what can be achieved using preclinical experimentation and analysis of biobank data.

## MATERIALS AND METHODS

### Peptides

GIP(1-42), GIP-TMR (*44*), and tirzepatide were obtained from WuXi AppTec (China).

### Plasmids and adenoviruses

Wild-type and single missense variant SNAP-GIPR plasmid constructs, all in the pcDNA5-FRT/TO vector, were obtained from Genewiz (UK). In these constructs, the endogenous GIPR signal peptide was removed and replaced with a SNAP_f_ tag with upstream signal peptide and a short SmBiT tag added at the C terminus. Halo-GIPR was generated in house as previously described (*44*). GIPR-YFP was a gift from D. Wootten (Monash University). Adenoviral SNAP-tagged human GIPR wild-type or variant constructs were generated by VectorBuilder.

### Cell culture

Adherent HEK293 (AD293) were cultured in Dulbecco’s modified Eagle’s medium (DMEM) supplemented with 10% fetal bovine serum (FBS) and 1% penicillin/streptomycin. INS-1 832/3 cells with CRISPR-Cas9 deletion of the endogenous *Gipr* gene were a gift from J. Naylor (AstraZeneca) (*28*), referred to herein as “INS-1 cells,” and were cultured in RPMI with 10% FBS and 1% penicillin/streptomycin.

### Islet isolation and culture

For ex vivo islet experiments, pancreatic islets were isolated from appropriate wild-type or *Gipr* ^−/−^ mice as previously described (*71*). Pancreata were infused via the common bile duct with RPMI 1640 medium (R8758, Sigma-Aldrich) containing collagenase (1 mg/ml) from *Clostridium histolyticum* (S1745602, Nordmark Biochemicals), dissected, and incubated in a water bath at 37°C for 10 min. Islets were subsequently washed and purified using a Histopaque gradient [Histopaque-1119, 11191 (Sigma-Aldrich) and Histopaque-1083, 10831 (Sigma-Aldrich)]. Isolated islets were allowed to recover overnight at 37°C in 5% CO_2_ in RPMI 1640 supplemented with 10% (v/v) FBS (F7524, Sigma-Aldrich) and 1% (v/v) penicillin/streptomycin solution (15070-063, Invitrogen).

### Assessment of surface GIPR expression by SNAP-tag labeling in cell lines

Twenty-four hours before the experiment, AD293 or INS-1 cells were reverse transfected with *GIPR* variant or wild-type plasmids using Lipofectamine 2000 in 96-well, clear-bottom, black microplates precoated with poly-d-lysine. All variants were tested in parallel on each occasion to control for transfection efficiency differences between experiments. Cells were then labeled using the cell-impermeant probe SNAP-Surface Alexa Fluor 647 (500 nM, New England Biolabs, USA) for 10 min at room temperature to label only surface GIPR. After washing three times with phosphate-buffered saline (PBS), cells underwent phase contrast and epifluorescence imaging in Krebs-Ringer bicarbonate-Hepes (KRBH) buffer [140 mM NaCl, 3.6 mM KCl, 1.5 mM CaCl_2_, 0.5 mM MgSO_4_, 0.5 mM NaH_2_PO_4_, 2 mM NaHCO_3_, and 10 mM Hepes, saturated with 95% O_2_/5% CO_2_ (pH 7.4)] using an automated wide-field microscope (Nikon Ti2E) with computer-controlled stage and focus lock. Several fields of view (FOVs) were acquired within each well using a 20X, 0.75–numerical aperture (NA) air objective. Surface GIPR fluorescence was quantified after flat-field correction using BaSiC (*72*) and segmenting using Phantast (*73*), which generates a mask based on the phase contrast images to demarcate cell versus noncell. Wide-field imaging of the surface-specific labeling achieved by SNAP-Surface probes means that surface expression can be established from whole-cell fluorescence, after subtracting nonspecific fluorescence from a separate well containing nontransfected cells processed in parallel from the same experiment. Data were normalized by dividing by the average specific signal across each experiment, or by the wild-type value, as indicated in the figure legends.

### Radioligand binding assay

Radioligand binding to the GIPR variant receptors was performed using the assay procedure described in (*59*) with some slight modification in the buffer. GIPR membrane protein and wheat germ agglutinin polyvinyl toluene scintillation proximity assay beads (WGA-PVT SPA beads, Revvity, catalog no. RPNQ0001) were incubated with a concentration-response curve of [^127^I]Tyr^10^-GIP(1-42)OH (CPC Scientific, San Jose, CA) and [^125^I]GIP(1-42)OH (Revvity, Waltham, MA) in 1.0 mM CaCl_2_ (Sigma-Aldrich, catalog no. 21115), 2.5 mM MgCl_2_ (Boston BioProducts, catalog no. BM670), 0.003% (v/v) Tween 20 (Roche Cat 11332465001), 0.1% (w/v) bacitracin (Thermo Scientific Chemicals, catalog no. J62432-14), 0.1% fatty acid–free human serum albumin (Sigma-Aldrich, catalog no. A3782), and 25 mM Hepes (Gibco, catalog no. 15630-080), pH to 7.4 with KOH, in a 200-μl assay volume. Human GIPR wild type, E354Q, E252D, and A207V used 6 μg of the membrane protein, 0.25 mg of the WGA-PVT SPA bead, and ∼0.1 nM [^125^I]GIP(1-42)OH. Human GIPR E288G, E288G+E354Q, and R101H used 12 μg of the membrane protein, 0.50 mg of the WGA-PVT SPA bead, and ∼0.3 nM [^125^I]GIP(1-42)OH. The assay was incubated overnight at 25°C and then centrifuged at 1000 rpm (Beckman Coulter, Avanti J-15R). Radioligand bound to the GIPR-expressing membrane (i.e., in close proximity to the WGA-PVT SPA bead) was detected using a PerkinElmer 2450 MicroPlate Counter. There was no significant specific binding detected in membranes prepared from untransfected HEK293 cells tested using either condition. Affinity and expression density values were obtained from homologous competition analysis of human [^127^I]Tyr^10^-GIP(1-42)OH versus human [^125^I]GIP(1-42)OH using the competitive binding equation one site homologous (GraphPad Prism version 10.4.1 for Windows).

### Assessment of total GIPR expression by SNAP-tag labeling

Experiments were performed and quantified as in the “Assessment of surface GIPR expression by SNAP-tag labeling in cell lines” section, except cells were labeled in parallel (separate wells) using 500 nM SNAP-Surface Alexa Fluor 647 or BG-OG (*29*) for 10 min at room temperature.

### Immunoblotting for total SNAP-GIPR

INS-1 cells were seeded in a 6-well plate and transfected with 2 μg of the *GIPR* variant or wild-type plasmids using Lipofectamine 2000 (Thermo Fisher Scientific) per the manufacturer’s instructions. All variants were tested in parallel on each occasion to control for transfection efficiency differences between experiments. Twenty-four hours posttransfection, the cells were lysed with 1X TNE lysis buffer (20 mM Tris, 150 mM NaCl, 1 mM EDTA, 1% NP-40, and protease and phosphatase inhibitor cocktails) for 10 min at 4°C followed by cell scraping and sonication (3X, 10 s each). The lysates were then centrifuged at 12000*g* for 10 min at 4°C. The supernatants were collected, fractionated by SDS–polyacrylamide gel electrophoresis (PAGE) in urea loading buffer [200 mM tris-HCl (pH 6.8), 5% (w/v) SDS, 8 M urea, 100 mM dithiothreitol, and 0.02% (w/v) bromophenol blue], and analyzed by Western blotting. SNAP-GIPR was detected with an anti–SNAP-tag rabbit polyclonal antibody (P9310S, New England Biolabs, RRID: AB_10631145, 1:1000) followed by goat anti-rabbit horseradish peroxidase (HRP) secondary (ab6721, Abcam, RRID: AB_955447, 1:2000). Poststripping, tubulin was labeled with anti–α-tubulin mouse monoclonal antibody (T5168, Sigma-Aldrich, RRID: AB_477579, 1:5000) followed by sheep anti-mouse HRP secondary antibody (ab6808, Abcam, RRID: AB_955441, 1:5000). Blots were developed with the Clarity Western enhanced chemiluminescence (ECL) substrate system (Bio-Rad) with the ChemiDoc Imaging System (Bio-Rad) and specific band densities quantified in Fiji.

### Prediction of variant thermal stability

Experimental GIPR structures (7RA3, 7RBT, and 7DTY) were obtained from the Protein Data Bank (PDB), and an AlphaFold 3–predicted full-length GIPR structure (AF-P48546-F1-model_v4) was obtained from the AlphaFold server (https://alphafold.com). After removing non-GIPR chains from the experimental structures, mCSM-membrane (*34*) was used to predict the thermostability effect of GIPR mutations present in the structures (the receptor C terminus is not included in experimental structures) as ∆∆*G*.

### Proteasomal inhibition

INS-1 cells were reverse transfected with the *GIPR* variant or wild-type plasmids in 96-well, clear-bottom, black microplates precoated with poly-d-lysine. All variants were tested in parallel on each occasion to control for transfection efficiency differences between experiments. Twelve hours posttransfection, the cells were treated with either vehicle or Proteasome Inhibitor I (Merck) for 4 hours. Following this, the cells were labeled with the cell-permeable SNAP-tag probe BG-OG (*29*) for 10 min at 37°C to label total SNAP. Cells were then imaged as in the “Assessment of surface GIPR expression by SNAP-tag labeling in cell lines” section to establish total GIPR labeling (surface and intracellular). Images were analyzed as in the “Assessment of surface GIPR expression by SNAP-tag labeling in cell lines” section.

### Constitutive and agonist-mediated internalization measured by reversible SNAP-tag labeling

Twenty-four hours before the experiment, AD293 or INS-1 cells were reverse transfected with *GIPR* variant or wild-type plasmids using Lipofectamine 2000 in 96-well, clear-bottom, black microplates precoated with poly-d-lysine. All variants were tested in parallel on each occasion to control for transfection efficiency differences between experiments. Cells were then labeled using the cleavable, cell-impermeant probe BG-SS-649 (a gift from New England Biolabs, USA) for 10 min at room temperature. Cells were then incubated for 60 min in serum-free medium containing 0.1% bovine serum albumin (BSA) at 37°C, with or without 100 nM GIP. Media were then removed, cells washed with PBS and then placed in TNE buffer (pH 8.6) for imaging. Cells were imaged as in the “Assessment of surface GIPR expression by SNAP-tag labeling in cell lines” section to establish total surface GIPR labeling, and after each well had been imaged, the cell-impermeant reducing agent Mesna (200 mM) was added to remove any surface BG-SS-649 bound to noninternalized GIPR. After 10 min, the well was reimaged again to detect remaining fluorescence from internalized GIPR. Images were analyzed as in the “Assessment of surface GIPR expression by SNAP-tag labeling in cell lines” section, with the residual specific fluorescence after Mesna treatment expressed relative to the pre-Mesna value to establish percentage internalization.

### Bafilomycin A1 experiments

Twenty-four hours before the experiment, AD293 or INS-1 cells were reverse transfected with *GIPR* variant or wild-type plasmids using Lipofectamine 2000 in 96-well, clear-bottom, black microplates precoated with poly-d-lysine. All variants were tested in parallel on each occasion to control for transfection efficiency differences between experiments. For AD293 cells, after 12 hours, 100 nM bafilomycin A1 or vehicle was added to the wells. A further 12 hours later, cells were labeled using SNAP-Surface Alexa Fluor 647 and imaged as described in the “Assessment of surface GIPR expression by SNAP-tag labeling in cell lines” section. For INS-1 cells, after 12 hours, 400 nM bafilomycin A1 or vehicle was added to the wells. After 4 hours of incubation with vehicle of bafilomycin A1, cells were labeled with SNAP-Surface Alexa Fluor 647 and imaged as described in the “Assessment of surface GIPR expression by SNAP-tag labeling in cell lines” section.

### Assessing for dominant negative effects by SNAP-tag and Halo-tag GIPR coexpression

Twenty-four hours before the experiment, AD293 cells were reverse cotransfected with SNAP-tagged *GIPR* variant or wild-type plasmids plus Halo-tagged wild-type GIPR in a 6:1 ratio using Lipofectamine 2000 in 96-well, clear-bottom, black microplates precoated with poly-d-lysine. All variants were tested in parallel on each occasion to control for transfection efficiency differences between experiments. After 24 hours, cells were dual-labeled with SNAP-Surface 488 (0.5 μM, New England Biolabs) and Halo-Alexa Fluor 660 (0.5 μM, Promega, UK) for 10 min at 37°C. After washing, cells were imaged as in the “Assessment of surface GIPR expression by SNAP-tag labeling in cell lines” section, and surface expression of SNAP-tag and Halo-tagged GIPR was quantified as in the “Assessment of surface GIPR expression by SNAP-tag labeling in cell lines” section.

### cAMP biochemical measurements

#### Single-concentration experiment

AD293 cells were reverse transfected with the *GIPR* variant or wild-type plasmids using Lipofectamine 2000 in 96-well microplates. All variants were tested in parallel on each occasion to control for transfection efficiency differences between experiments. After 24 hours, cells were treated with GIP (100 pM or 100 nM) or 500 μM 3-isobutyl-1-methylxanthine (IBMX) in serum-free medium containing 0.1% BSA for 30 min at 37°C, before terminating the reaction by adding lysis buffer and measuring cellular cAMP by HTRF (cAMP Dynamic 2 assay, Revvity). IBMX was not used in the assays using GIP. cAMP concentration measured from untransfected cells was subtracted to isolate the receptor/agonist-specific response.

#### Concentration-response experiments

(Imperial College London): 24 hours before the experiment, AD293 cells were reverse transfected with the *GIPR* variant or wild-type plasmids using Lipofectamine 2000 in 12-well plates. All variants were tested in parallel on each occasion to control for transfection efficiency differences between experiments. Cells were then treated in suspension with a range of concentrations of GIP or tirzepatide in serum-free medium containing 0.1% BSA and 250 μM IBMX for 30 min at 37°C, before terminating the reaction by adding lysis buffer and measuring cellular cAMP by HTRF. Data were fit using a three-parameter logistic model using GraphPad Prism 10.

#### Concentration-response experiments

(Eli Lilly): HEK293 cells transiently expressing the human GIPR (NP_000155) or the variants described herein were evaluated by measuring the accumulation of intracellular cAMP using the Gs Dynamic Assay (PerkinElmer), as previously described (*59*). The assay medium was DMEM (Thermo Fisher Scientific) containing 2 mM GlutaMAX (Thermo Fisher Scientific), 20 mM Hepes, 0.1% (w/v) bovine casein (MilliporeSigma), and 250 μM IBMX. Cells were added to treatment plates and incubated at 37°C for 30 min and then lysed in the presence of a d2-labeled cAMP competitor conjugate and cryptate-conjugated detection antibody. Time-resolved fluorescence was measured with a Pherastar FSX reader (BMG Labtech), and data were analyzed by standard ratio methods. Normalized values were fit to the four-parameter logistic model using the GraphPad Prism 7 software.

### Mini-G_s_ recruitment

AD293 cells were reverse transfected with the *GIPR* variant or wild-type plasmids plus mini-Gs-LgBiT in a 1:1 ratio using Lipofectamine 2000 in 96-well white-bottom microplates precoated with 0.01% poly-d-lysine. All variants were tested in parallel on each occasion to control for transfection efficiency differences between experiments. After 24 hours, media were removed, cells washed once, and HBSS (Hanks’ balanced salt solution) containing furimazine (Promega, UK) at a 1:100 dilution was added. Total luminescence was recorded at regular intervals before and after addition of 100 nM GIP or vehicle over a 10-min stimulation using a Flexstation 3 plate reader at 37°C. Responses were quantified by as fractional change from baseline with subtraction of the response to vehicle to account for substrate depletion, from which the AUC (area under the curve) was calculated and scaled to the average response obtained from each experiment.

### cAMP imaging

AD293 cells were reverse transfected with the *GIPR* variant or wild-type plasmids using Lipofectamine 2000 and cotransduced with the cADDis Green Up BacMam (10 μl per well, Montana Molecular, USA), in 96-well, poly-d-lysine–coated microplates. All variants were tested in parallel on each occasion to control for transfection efficiency differences between experiments. After 24 hours, cells were labeled using 500 nM SNAP-Surface Alexa Fluor 647 for 10 min at room temperature. Cells were washed three times and then imaged in KRBH buffer containing 6 mM glucose and 0.1% BSA. Imaging was performed at 37°C by wide-field microscopy using the same microscope as in the “Assessment of surface GIPR expression by SNAP-tag labeling in cell lines” section, with 9 FOVs for both cADDis and SNAP-Surface fluorescence acquired from each well using a 10X, 0.45 NA air objective. For steady-state assays, images were acquired at baseline, 10 min after addition of 100 pM or 100 nM GIP, and again 10 min after addition of 500 μM IBMX and 50 μM forskolin (FSK). Flat-field correction was performed using BaSiC (*72*), drift correction was performed using SIFT, and a maximum intensity projection of the cADDis channel was used to segment individual cells. cAMP responses were determined by quantifying the cADDis fluorescence intensity for each cell at each time point. Nonviable cells or noncell objects were excluded according to criteria for allowable cell IBMX/FSK responses. The GIP effect for each cell was quantified by double normalizing the cADDis signal to baseline and then to IBMX/FSK. The cADDis mask was then used to quantify the SNAP-Surface signal from each cell. Wild-type SNAP-Surface readings were split into deciles, and these deciles were subsequently used to categorize variant-transfected cells to match against the wild type. After trimming the top and bottom deciles, the mean was taken of each decile mean for surface expression level or cAMP response, which achieved matched expression levels by effectively applying an inverse weighting to counteract differences in cell number in each decile for different variants. Ensemble expression levels or cAMP responses were calculated without regard to deciles. Variant cADDis responses were also analyzed within their own surface expression deciles.

### Assessment of surface GIPR expression by SNAP-tag labeling in pancreatic islets

Islets were purified from *Gipr*^−/−^ mice as described in the “Islet isolation and culture” section. Islets were transduced with adenoviral wild-type or variant SNAP-tagged *GIPR* constructs (VectorBuilder) per the manufacturer’s instructions. All variants were tested in parallel on each occasion to control for transduction efficiency. Twenty-four hours posttransduction, whole, intact islets were labeled with SNAP-Surface Alexa Fluor 647 for 15 min at 37°C. Following labeling, the islets were placed in a drop of PBS in the center of a glass-bottom MatTek dish and imaged on a Zeiss LSM 780 inverted confocal microscope fitted with a 63X/1.4 NA oil immersion objective. Receptor surface levels were quantified in Fiji using a macro using full width at half maximum (FWHM) designed by the Facility for Imaging and Light Microscopy (FILM) at Imperial College London and expressed relative to wild-type GIPR. In additional experiments, islets transduced as above and labeled with SNAP-Surface-647 were fixed in 4% paraformaldehyde, permeabilized in 0.5% (v/v) Triton X-100 in PBS (15 min), washed once with PBS and incubated in blocking buffer (PBS and 1% BSA) for 1 hour, and incubated overnight at 4°C in primary antibodies against insulin (1:50; Dako IR002, Agilent Technologies) and glucagon (1:500; G2654, Sigma-Aldrich) in blocking buffer. Islets were then washed three times in PBS and incubated at room temperature with secondary anti–guinea pig Alexa Fluor 488 and anti-mouse Alexa Fluor 568 (both at 1:500; Thermo Fisher Scientific) for 1 hour, washed three times, and images/analyzed as above.

### Hormone secretion assays from pancreatic islets

Isolated islets used for insulin secretion assays were treated in 1.5-ml microcentrifuge tubes. Eight-ten islets were used per well with two-three technical replicates per condition. Islets were preincubated for 1 hour in KRBH buffer [140 mM NaCl, 3.6 mM KCl, 1.5 mM CaCl_2_, 0.5 mM MgSO_4_, 0.5 mM NaH_2_PO_4_, 2 mM NaHCO_3_, and 10 mM Hepes, saturated with 95% O_2_/5% CO_2_ (pH 7.4)] containing 0.1% (w/v) BSA and 3 or 11 mM glucose for insulin or glucagon secretion, respectively. This was followed by incubation with 11 or 0.5 mM glucose ±100 nM GIP in KRBH buffer for insulin or glucagon secretion, respectively, in a shaking 37°C water bath (80 rpm) for 30 min. At the end of the treatment, supernatants containing the secreted insulin or glucagon were collected, centrifuged at 1000*g* for 5 min, and transferred to fresh tubes. To determine total insulin or glucagon contents, islets were lysed using acidic ethanol [75% (v/v) ethanol and 1.5 mM HCl]. The lysates were sonicated 3× for 10 s in a water bath and centrifuged at 10,000*g* for 10 min, and the supernatants were collected. The samples were stored at −20°C until the insulin or glucagon concentrations were determined using an Insulin Ultra-Sensitive HTRF Assay kit (62IN2PEG, Revvity) or an HTRF Glucagon Detection Kit (62CGLPEG, Revvity) respectively, according to the manufacturer’s instructions. GraphPad Prism 9.0 was used for the generation of the standard curve and sample concentration extrapolation. The total insulin or glucagon content was calculated by adding the secreted insulin to the insulin content of the lysates, and secretion was reported as the percentage release of total. The percent secretion of islets treated with agonist was normalized to that of islets treated with vehicle and reported as a fold change.

### Statistics for wet laboratory experiments

The average of technical replicates was considered as one biological replicate for in vitro experiments. Experiments were performed with all conditions tested in parallel to allow matched analyses, which were performed using Prism 10.5.1 (GraphPad Software). Specific statistical tests, including corrections for multiple comparisons, are indicated in the figure legends. Statistical significance was inferred when p < 0.05. Data are represented as means ± SEM, or with 95% confidence intervals where specifically indicated, and some figures include experimental replicates as individual data points.

### MD simulations

The membrane-protein system was prepared using a custom Desmond System Builder file optimized for OPM-aligned membrane-bound proteins. The protein-lipid system was embedded in a preequilibrated POPC bilayer with 15-Å buffers in each dimension to ensure adequate solvation and spatial separation. The SPC water model was used to solvate the system, and a 0.15 M NaCl concentration was added to approximate physiological ionic strength. Counter-ions were included to neutralize the system. Overlapping crystallographic water molecules were removed, and solvent van der Waals radii were scaled by 0.75 to reduce steric clashes. The OPLS4-based S-OPLS force field was assigned to all system components. Hydrogen mass repartitioning was enabled to support the use of larger integration time steps in subsequent MD simulations. Noncrystallographic water molecules were assigned to an energy group to enable selective control during membrane relaxation and equilibration. System construction was performed using Schrodinger’s Desmond preparation framework. All MD simulations were performed under periodic boundary conditions with temperature maintained at 300 K unless otherwise stated using the Desmond simulation engine (*74*) within the (*75*) software suite. A custom membrane protein equilibration and relaxation protocol was used. This protocol is designed to gradually relax steric clashes and artifacts associated with protein crystallography and membrane packing, especially following system assembly in a lipid bilayer environment. The system underwent a staged equilibration process, comprising five distinct phases:

1) Initial Brownian dynamics (NVT, 10 K, 50 ps): A short Brownian dynamics simulation at 10 K and constant volume (NVT ensemble) was used to minimize high-energy contacts. Solute heavy atoms were harmonically restrained (50 kcal/mol per square angstrom). A small timestep (0.001 to 0.003 ps) and the Brownie integrator were used to accommodate potentially large initial forces.

2) High-pressure packing (NPT, 100 K, 20 ps): The system was then equilibrated at 100 K and high pressure (1000 atm) using another Brownian dynamics step in the NPT ensemble to enhance membrane-protein packing. Harmonic restraints were applied anisotropically to membrane heavy atoms in the *z* dimension and to solute heavy atoms (20 kcal/mol per square angstrom). A GaussianBarrier plug-in was applied to prevent water intrusion between the membrane and protein, implementing two Gaussian repulsive potentials centered at ±10 Å along the *z* axis (σ = 0.5 Å, *A* = 10 kcal/mol) to act only on water molecules previously reassigned to a distinct energy group.

3) Further packing under NPgT (100 K, 100 ps): A 100-ps simulation at 100 K and 1000 atm was conducted using the MTK integrator in the NPgT ensemble, allowing anisotropic pressure coupling in *x*-*y* versus *z*. Restraints were weakened (2 kcal/mol per square angstrom on lipid headgroups and 10 kcal/mol per square angstrom on solute heavy atoms), and Gaussian barriers were retained.

4) Gradual heating and pressure reduction (NPgT, 100 → 300 K, 150 ps): The system was then heated from 100 to 300 K over 150 ps using an annealing schedule. Restraints remained as above, and the barostat and thermostat coupling constants were set to 2.0 and 0.1 ps, respectively. A simplified GaussianForce plug-in (σ = 5 Å, *A* = 2 kcal/mol centered at *z* = 0) replaced the dual-barrier setup to continue discouraging water intrusion.

5) Production run (NPgT, 300 K): After equilibration, restraints and Gaussian barriers were removed, and production simulations were run under NPgT conditions at 300 K and 1 atm. Surface tension was maintained in the *x*-*y* plane, with anisotropic pressure scaling. A timestep scheme of 2 fs (with multiple timestep RESPA integration for bonded and nonbonded interactions) was used during standard MD runs, except during Brownian dynamics stages. Nosé-Hoover thermostats and Martyna-Tobias-Klein barostats were used to maintain temperature and pressure, respectively (*76*, *77*). Final simulations times were ∼1 μs. Approximately 750 ns of production trajectory data was collected at appropriate intervals for downstream analysis. Protein-protein interactions, kink angles, and clustering analyses were all performed using in-house custom workflows.

### Exome-wide association study: Ethics and study approval

The UKBB operates under approval from the North West Multi-centre Research Ethics Committee (REC reference 13/NW/0157) as a Research Tissue Bank (RTB), with informed consent obtained from all participants. This approval allows researchers to conduct studies without requiring separate ethical clearance as they are covered by the RTB approval. Initially granted in 2011, this approval is renewed every 5 years, with successful renewals completed in 2016 and 2021. The work described for this analysis was approved by the UKBB through resource application number 9905.

### Exome-wide association study: Data and quality control

We accessed WES data on up to 469,835 participants from the UKBB study (470k WES release, July 2022) (*78*). We analyzed data on 465,797 individuals after excluding samples with excess heterozygosity, autosomal variant missingness on genotyping arrays ≥ 5%, or were not included in the subset of phased samples as defined by Bycroft *et al.* (*79*). For our European-only sample (*n* = 434,438 individuals), we excluded participants who were not of broadly European genetic ancestry. For each outcome, further exclusions were implemented due to incomplete phenotype data (see table S6). Continuous outcome traits were BMI and HbA1c (excluding diabetes cases). T2D was the only binary outcome (defined in table S6). Outcome data were taken from the first UKBB study visit, unless otherwise specified in table S6. Rare variant burden testing was performed on both the European-only and the full multiethnic UKBB samples. As results were consistent in both strata and statistical power was enhanced in the latter by its larger sample size and by incorporating rare variants present in non-Europeans, single-variant analysis was conducted only on the full multiethnic UKBB sample. All data processing and analyses were performed within the UKBB Research Analysis Platform (RAP; https://ukbiobank.dnanexus.com/). This is a cloud-based computing environment and the central repository for UKBB WES and phenotype data. WES data are stored as population-level variant call format (VCF) files, aligned to GRCh38. In addition to the processing applied to the released data, as documented by Backman *et al.* (*78*), we performed further quality control measures as described by Gardner *et al.* (*80*) and detailed in the MRC-EPID WES pipeline (https://github.com/mrcepid-rap/).

### Exome-wide association study: Single-variant and burden association tests

We conducted burden tests by collapsing rare (MAF < 1%) *GIPR* variants with similar experimental functional consequences, specifically their 100 pM GIP cAMP responses and levels of surface expression in AD293 cells. We assigned variants to reduced, neutral, and increased response groups, with “reduced” defined as a statistically significant reduction of at least 50% below that of the wild-type receptor. The common missense variant, E354Q, was excluded from burden tests due to its high population prevalence (>19% in UKBB) (*81*). The remaining *GIPR* variants were grouped on the basis of their impact on surface expression into two categories: (i) 11 variants with reduced surface expression and (ii) 15 variants with a neutral impact on expression compared to the wild type. The I378M variant was the only one to exhibit increased surface expression and, therefore, could not be tested in a group for burden analysis due to the low number of carriers. Similarly, *GIPR* variants were also categorized on the basis of their cAMP response to GIP stimulation into three categories: (i) 14 variants with reduced cAMP response, (ii) 11 variants with a neutral impact on cAMP response, and (iii) 2 variants with increased cAMP response. We performed rare variant burden association testing using two statistical methods: STAAR (“variant-Set Test for Association using Annotation infoRmation”) (*46*) and a generalized linear regression model as implemented in the Python package “Statsmodels.” STAAR effectively accounts for population structure and relatedness, so it was considered to be particularly applicable to analyses of the full multiethnic sample where population differences are potential confounders. The generalized linear model is widely used but it is less robust for analyzing rare variants, especially when population structure and relatedness are significant factors, so it was included as a sensitivity measure. We also performed single-variant association testing using BOLT-LMM, a mixed-model algorithm (v2.4.1) (*47*). BOLT-LMM calculates the genetic relationship matrix (GRM), or kinship matrix, to account for potential confounding by population stratification and hidden relatedness. We applied BOLT-LMM in the customized applet for each outcome, with default settings and the “ImmInfOnly” flag, which limits BOLT-LMM to an “infinitesimal” mixed model. BOLT-LMM requires two main data inputs: (i) genotypes from genotyping arrays, filtered for variants with a minor allele count of >100, to build a null model, and (ii) a broader set of imputed variants for association testing. For the first input, we accessed UKBB array genotype data available on the RAP, in a sample identical to that used for rare variant testing. For the latter, we included all 27,040,378 single markers in the input to BOLT-LMM, regardless of filtering status. Single variant–level BOLT association summary statistics were then extracted for the 30 functionally characterized variants. Significance thresholds were established using Bonferroni correction for multiple testing of 28 variants in the two main uncorrelated phenotypes (BMI and HbA1c) (i.e., *P* value < 9 × 10^−4^ was considered to be statistically significant, and it was calculated as 0.05/[28 variants x 2 phenotypes]) (*82*). All analyses were performed using bespoke applets designed for the RAP as described in the MRC-EPID WES pipeline (https://github.com/mrcepid-rap/), controlled for sex, age, age^2^, the first 10 genetic ancestral principal components (PCs) as calculated by Bycroft *et al.* (*79*), and WES release batch (50k, 200k, and 450k).
