# Supplementary material for: Glycemic and bodyweight effects of GIPR coding variation reflect differences in surface expression and intrinsic functional impairment
Source: Sci Adv. 2026 Sep 4;12(36):eaec3372. doi: 10.1126/sciadv.aec3372 (PMC13544220; doi:10.1126/sciadv.aec3372)
Supplement: Supplementary file 1 [file sciadv.aec3372_sm.pdf]

Science Advances
AAAS

Supplementary Materials for

Glycemic and bodyweight effects of *GIPR* coding variation reflect differences in surface expression and intrinsic functional impairment

Yusman Manchanda *et al.*

Corresponding author: Alejandra Tomas, a.tomas-catala@imperial.ac.uk; Ben Jones, ben.jones@imperial.ac.uk

Sci. Adv. **12**, eaec3372 (2026)
DOI: 10.1126/sciadv.aec3372

The PDF file includes:

Figs. S1 to S7
Tables S1, S4 and S5
Legends for tables S2, S3 and S6

Other Supplementary Material for this manuscript includes the following:

Tables S2, S3 and S6
Data file S1

Supplementary Figure 1

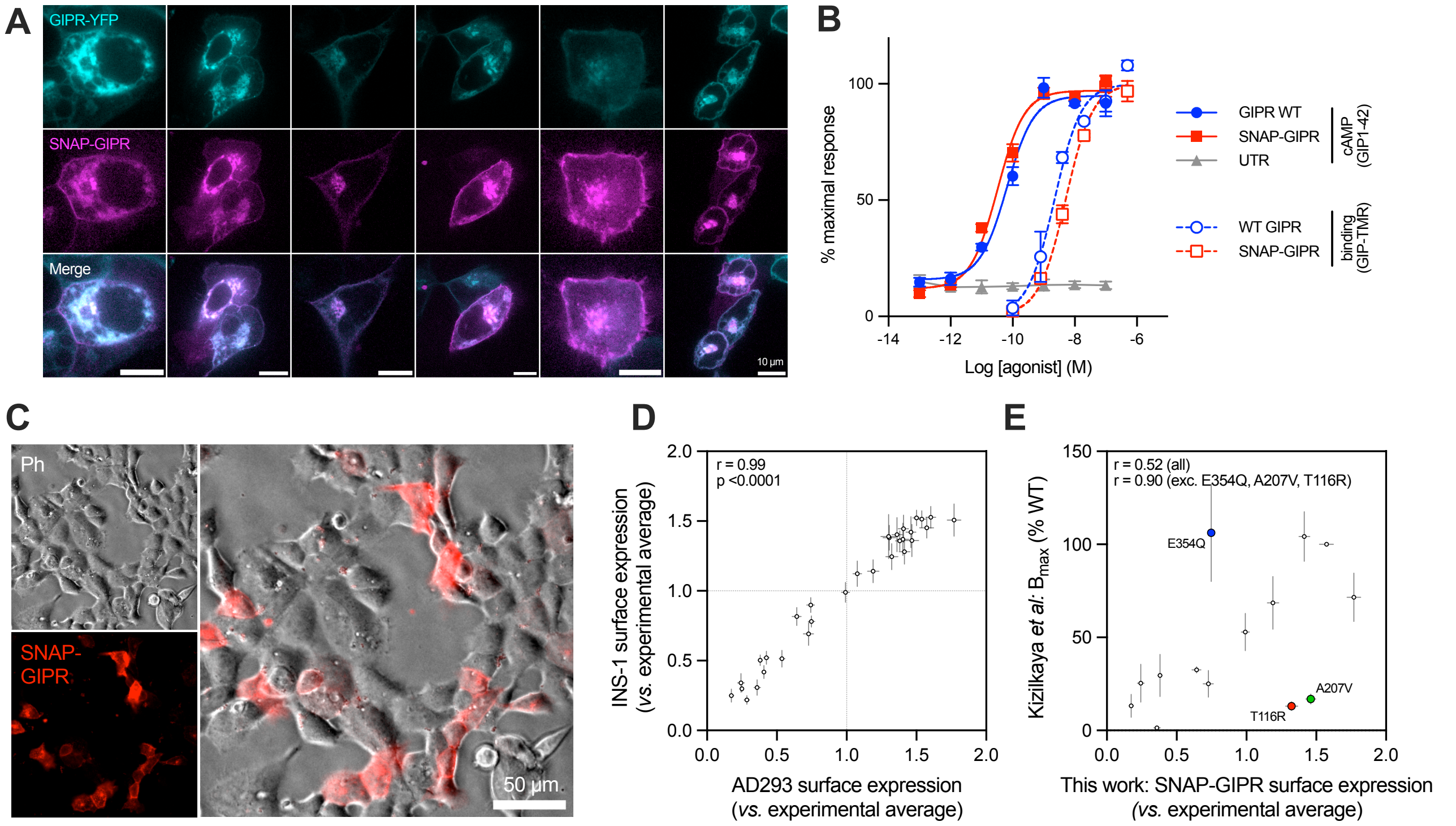

Supplementary Figure 1. GIPR variant surface expression. (**A**) Example images of AD293 cells co-transfected in a 1:1 ratio with GIPR-YFP and SNAP-GIPR, with labelling using SNAP-Cell-SiR prior to imaging to label whole cell SNAP-GIPR. Scale bars = 10 µm. (**B**) cAMP responses to GIP(1-42) (*n*=4) and binding of GIP-TMR (*n*=2) to AD293 cells transfected with wild-type (WT; untagged) GIPR or SNAP-tagged GIPR, *n*=4. (**C**) Example image from high content analysis of GIPR surface expression. Scale bar = 50 µm. (**D**) Comparison of GIPR variant surface expression in AD293 and INS-1 cells, using data from Figure 1C and 1D. (**E**) Comparison of AD293 surface expression levels from Figure 1C and the B_{max} for ^{125}I-GIP measured by Kizilkaya *et al.* (*21*). All data represented as mean ± SEM.

Supplementary Figure 2

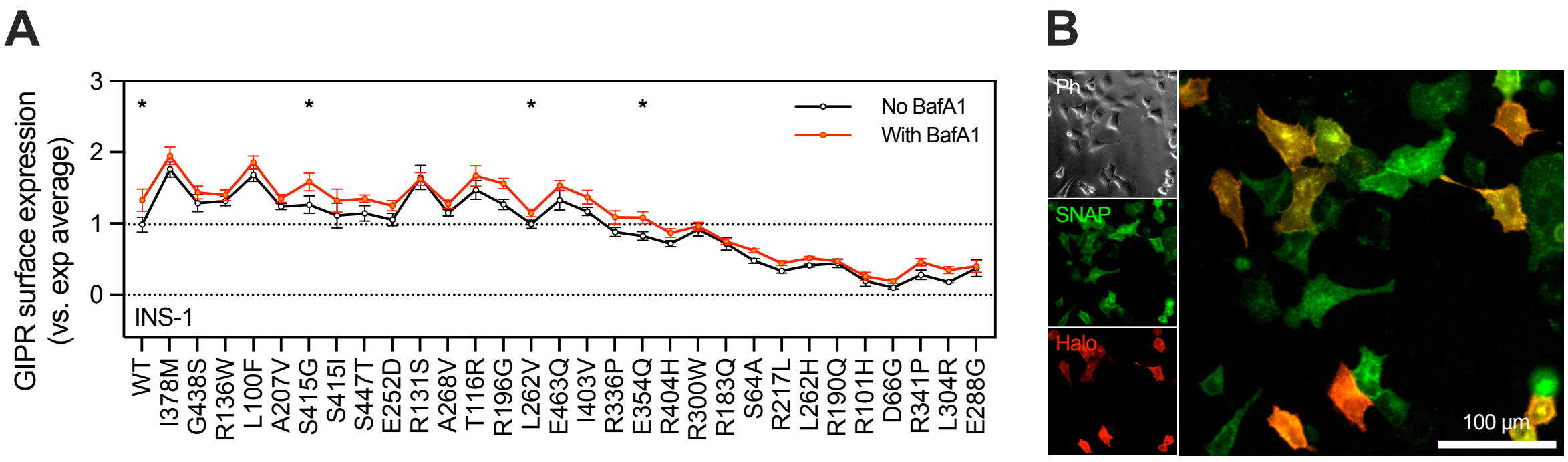

Supplementary Figure 2. Investigating the mechanism of altered surface expression with GIPR variants. (**A**) Effect of bafilomycin A1 treatment (4 hours, 400 mM) on relative surface expression of SNAP-GIPR in AD293 cells, *n*=8, scaled to experimental average without PSI, and compared using two-way matched ANOVA with Sidak's test with/without bafilomycin A1. (**B**) Representative image from high content analysis of SNAP- and Halo-GIPR co-expression with surface-specific dual colour labelling. Scale bar = 100 µm. *p<0.05 using indicated statistical test. All data represented as mean ± SEM.

Supplementary Figure 3

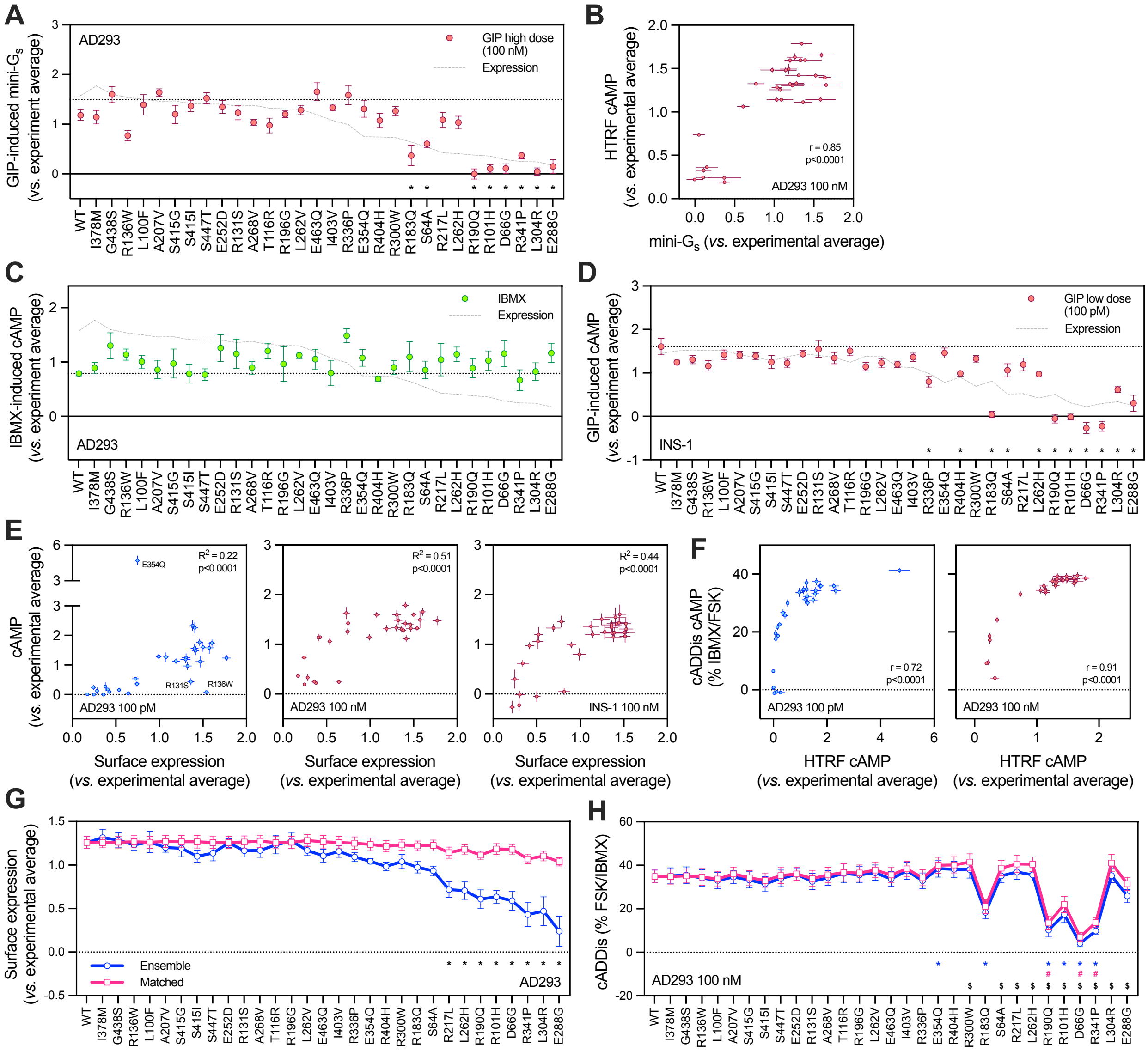

Supplementary Figure 3. Cyclic AMP signalling. (**A**) mini-G$_s$ recruitment responses to 100 nM GIP measured by nanoBiT assay in AD293 cells, *n*=7, with scaling to the experimental average and statistical comparisons by one-way matched ANOVA with Dunnett's test *versus* wild-type. (**B**) The relationship between mini-G$_s$ (Supplementary Figure 3A) and 100 nM cAMP HTRF results (from Figure 3B). (**C**) cAMP responses measured by HTRF in the presence of 500 µM IBMX but no GIP in AD293 cells, *n*=4. Results are scaled to the experimental average and compared by two-way matched ANOVA with Dunnett's test *versus* wild-type. The dotted line shows the wild-type mean and the dashed line shows the surface expression level from Figure 1C. (**D**) As for (C) but showing responses to 100 nM GIP in INS-1 cells, *n*=8. (**E**) The relationship between surface expression levels of wild-type and variant GIPR in AD293 and INS-1 cells (from Figure 1C and 1C) and the corresponding HTRF cAMP response to 100 pM or 100 nM GIP. (**F**) The relationship between HTRF and cADDis biosensor measured cAMP responses. (**G**) and (**H**) show equivalent data and analysis to data from Figure 3E and 3F, except with higher GIP concentration (100 nM). *p<0.05 (variant *versus* wild-type from ensemble cells), #p<0.05 (variant *versus* wild-type from expression-matched cells), $p<0.05 (ensemble *versus* expression-matched cells). All data represented as mean ± SEM.

Supplementary Figure 4

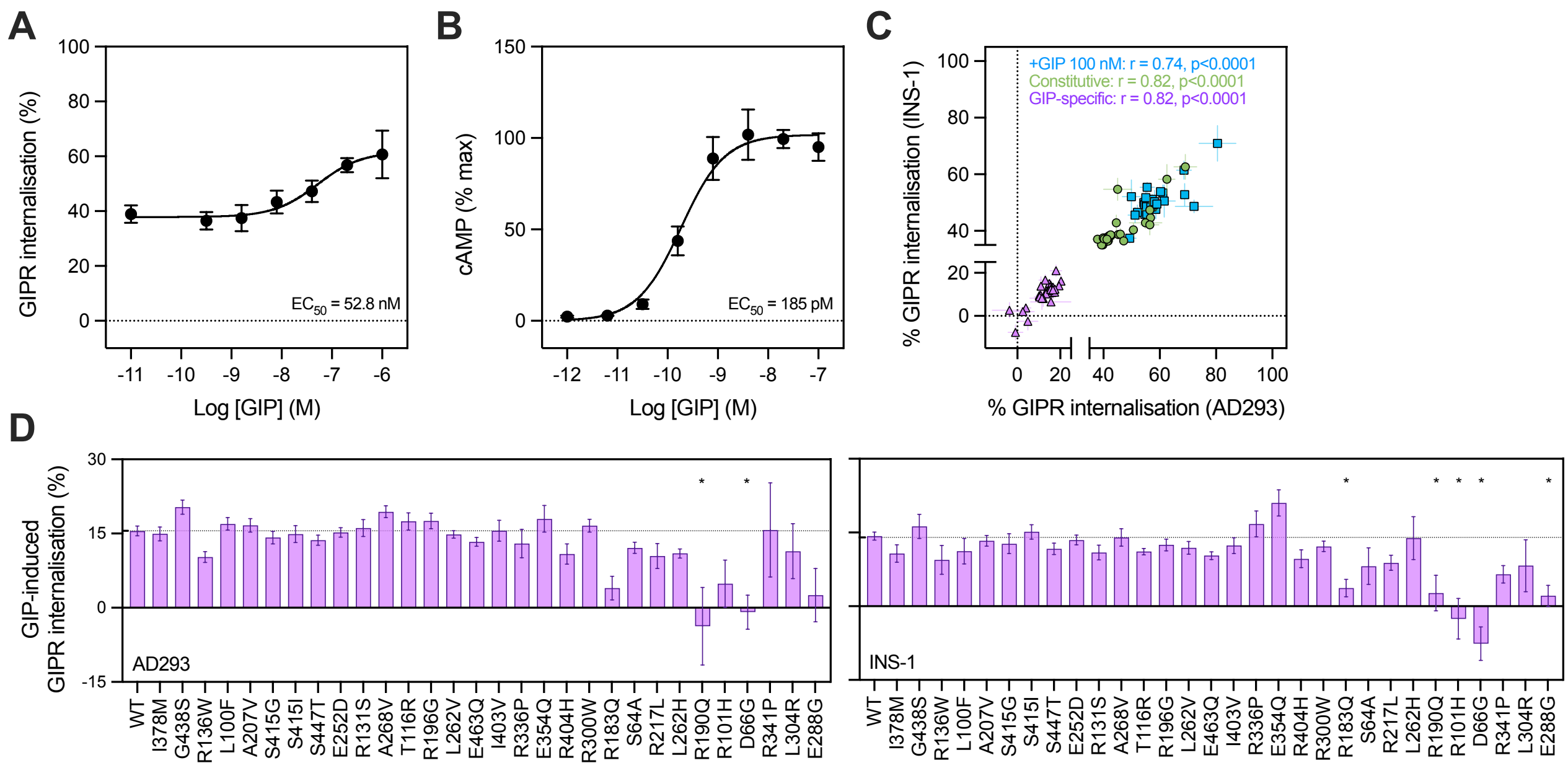

Supplementary Figure 4. GIPR internalisation. (**A**) Wild-type SNAP-GIPR percentage internalisation concentration response, *n*=5. (**B**) Wild-type SNAP-GIPR cAMP concentration response, *n*=5. (**C**) Correlation between AD293 and INS-1 internalisation percentages. (**d**) Agonist-specific percentage GIPR internalisation in AD293 and INS-1 measured by subtracting vehicle from GIP response in Figure 4B and 4C, with comparison by one-way matched ANOVA with Dunnett's test *versus* wild-type. *p<0.05 using indicated statistical test. All data represented as mean ± SEM.

Supplementary Figure 5

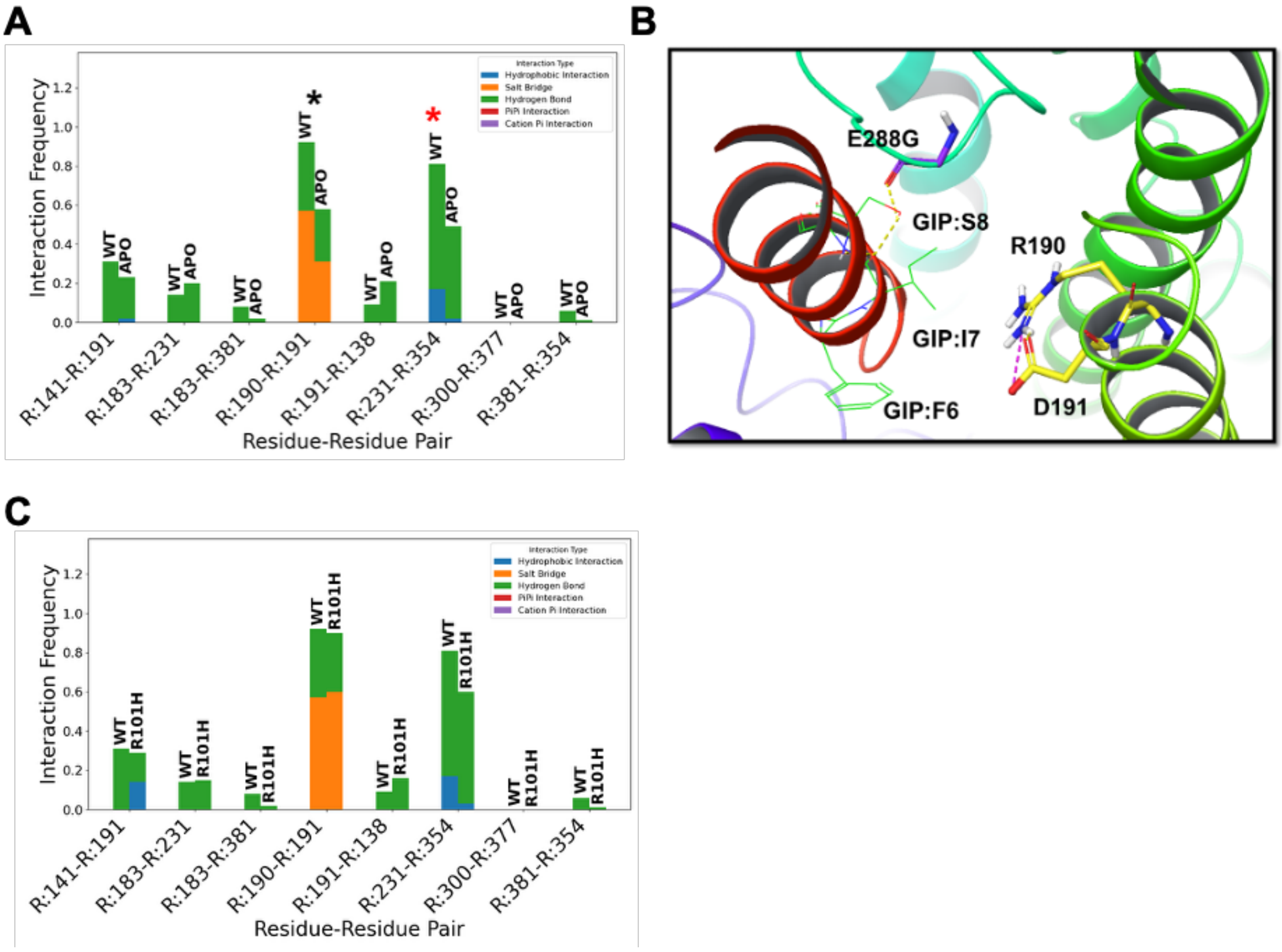

Supplementary Figure 5. Molecular dynamics simulations. (**A**) Wildtype receptor in the GIP bound and APO state. The intrahelical interaction in TM2 between neighbouring residues R190 and D191 is significantly enhanced in the presence of GIP. This suggests the setup of this local network, as well as TM4:Y231 TM6:E354 interhelical network is critical for GIP binding. (**B**) Representation of GIP (red ribbons) movement within the binding region in the presence of the E288G mutation. Interaction residues shown as stick representation. Hydrogen bonds are shown as yellow dashed lines and charge-charge interactions are shown as magenta dashed lines. (**C**) Mutation R101H show minor disruption of long-range protein-protein interactions at TM4-TM6. While the R101H mutation does not disrupt local orthosteric interactions typically observed in the presence of GIP, there is a small consistent long-range effect. These data together with other computational mutants suggest the TM6 E354 – TM4:Y231 inter-helical bundle interaction is very sensitive to changes in the receptor sequence. Since E354 is on TM6, directly implicated in signal transmission through the TM6 kink, even long distance (both sequentially and spatially) could affect G-protein engagement.

Supplementary Figure 6

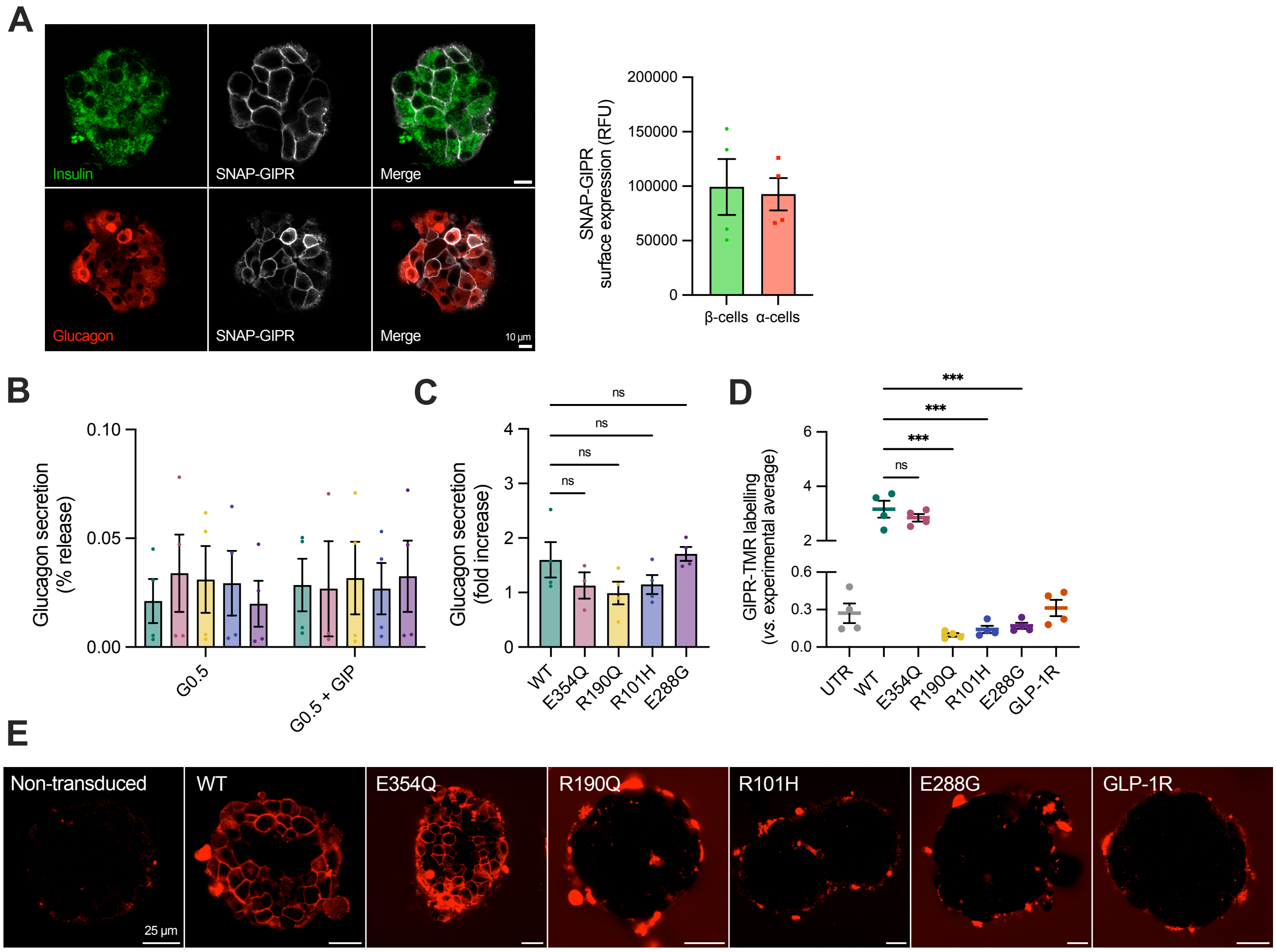

Supplementary Figure 6. Effects in pancreatic islets. (**A**) Co-staining of insulin or glucagon with SNAP-GIPR transduced into *Gipr* $^{-/-}$ islets with quantification of SNAP-labelling for individual islets from a single preparation of several islets. (**B**) Release of glucagon from *Gipr*$^{-/-}$ mouse islets transduced with GIPR adenoviruses at 0.5 mM glucose (G0.5), with and without 1000 nM GIP, 30-minute stimulation, *n*=4. Wild-type and variant responses compared by two-way matched ANOVA with Dunnett's test. (**C**) Data from (B) analysed as GIP-induced fold-change, with comparison using one-way matched ANOVA with Dunnett's test. (**D**) Quantification of GIP-TMR labelling (1000 nM, 30 minutes) in wild-type mouse islets transduced with SNAP-GIPR adenoviruses, *n*=4 repeats, comparison by one-way matched ANOVA with Dunnett's test *versus* wild-type. (**E**) Representative images for data shown in (D); scale bars = 25 µm. ***p<0.0001 using indicated statistical test. All numerical data represented as mean ± SEM with individual data points.

Supplementary Figure 7

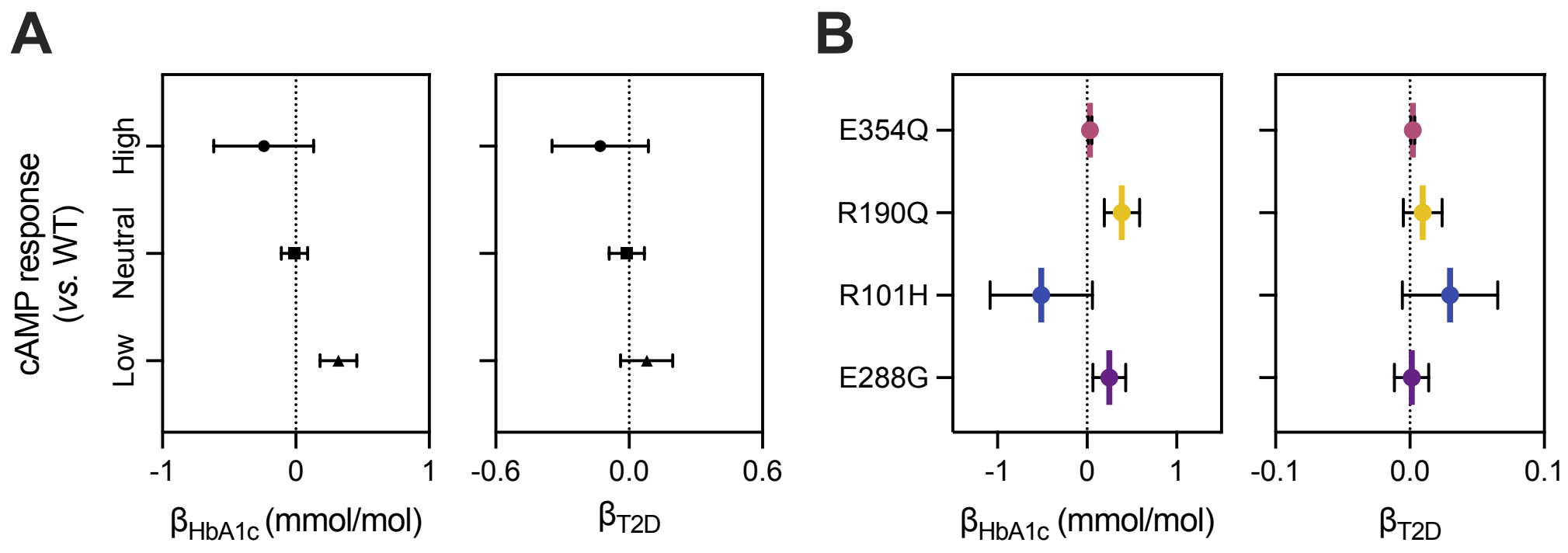

Supplementary Figure 7. Genetic association analysis. (**A**) Results of burden test analysis, showing effects of *GIPR* variants grouped by cAMP response category on HbA1c and T2D risk, without adjustment for BMI. (**B**) Results of selected single variant association analyses, showing effects of E354Q, R190Q, R101H and E288G on HbA1c and T2D risk, without adjustment for BMI. Data are represented as mean ± 95% confidence intervals.

Supplementary Table 1. Human [^{125}I]GIP(1-42)OH binding to human GIP-R receptor mutants expressed in HEK-293 cells.

**Receptor**	**K_d [a] (nM) (SEM, n)**	**B_{max} [b] (fmol/mg protein) ± SEM (n)**
hGIPR WT	0.383 (0.038, 5)	1340 ± 280 (5)
hGIPR (E354Q)	0.327 (0.045, 4)	1150 ± 130 (4)
hGIPR (E252D)	0.422 (0.038, 5)	1200 ± 210 (5)
hGIPR (A207V)	0.467 (0.069, 3)	1680 ± 30 (3)
hGIPR (E288G) [c]	10.1 (7.5, 5)	2870 ± 1290 (5)
hGIPR (E288G + E354Q) [c]	23.2 (7.6, 5)	4130 ± 1730 (5)
hGIPR (R101H) [c]	2.72 (1.67, 6)	927 ± 397 (6)

[a] K_d values are reported as the geometric mean with the SEM and the number of independent experiments in parentheses.
[b] B_{max} values are the arithmetic mean ± SEM with the number of independent experiments in parentheses.
[c] Denotes that, though expression was seen, due to the lower affinity for human [^{125}I]GIP(1-42)OH at these three mutant receptors an accurate K_d and B_{max} were difficult to obtain using this radioligand. The bottom of the fitted [^{127}I]Tyr^{10}-GIP(1-42)OH curve was used to define nonspecific binding for these three mutant receptors. Nonspecific binding was defined by 100 nM or 1 µM GIP(1-42)OH for the remaining mutant and wild type human GIPR.

Supplementary Table 2. Results from all burden testing analysis models, related to Figure 7a. This table is provided in Excel format, uploaded separately.

Supplementary Table 3. Results from single-variant association testing analysis, related to Figure 7b. This table is provided in Excel format, uploaded separately.

Supplementary Table 4. Summary of surface expression, pharmacology, hormone secretion, and human phenotypic effects of key variants E354Q, E288G, R190Q and R101H, relative to wild-type.

	E354Q	R190Q	R101H	E288G
Surface Expression	↓	↓↓↓	↓↓↓	↓↓↓
Proteasomal degradation	-	↑	↑	↑
cAMP (low [GIP])	↑↑	↓↓	↓↓	↓↓
cAMP (high [GIP])	-	↓↓	↓↓	↓↓
Insulin secretion (fold)	↓↓	↓↓↓	↓	↓
Glucagon secretion (fold)	↓	↓	-	↓
BMI	↓	↓↓	↓	↓↓
HbA1c (BMI adjusted)	↑	↑↑	↓	↑↑
T2D risk	↑	↑	↑	↑

Supplementary Table 5. hGIP(1-42)NH_2 and tirzepatide cAMP responses in cells expressing untagged human GIP-R and variants. Assays were performed in the presence of 0.1% casein.

	**cAMP Human GIP(1-42)NH_2 in human GIP-R receptor mutants expressed in HEK-293 cells.**		**cAMP Tirzepatide in human GIP-R receptor mutants expressed in HEK-293 cells.**	
**Receptor**	**EC_{50} nM (SEM, n)**	**E_{max} % (SEM, n)**	**EC_{50} nM (SEM, n)**	**E_{max} % (SEM, n)**
hGIPR WT	0.00151 (.0001,4)	96.4 (0.82, 4)	0.00148 (0.0002, 4)	90.6 (0.89, 4)
hGIPR (E354Q)	0.000383 (0.00003, 2)	98.7 (0.275, 2)	0.000821 (0.0001, 2)	104 (2.50, 2)
hGIPR (E252D)	0.00189 (0.0002, 2)	104 (1.15, 2)	0.00197 (0.0001, 2)	97.1 (5.18, 2)
hGIPR (A207V)	0.00160 (0.0002, 2)	106 (9.98, 2)	0.00180 (0.0003, 2)	94.5 (2.86, 2)
hGIPR (E288G) [c]	0.338 (0.0592, 2)	101 (2.63, 2)	0.191 (0.0194, 2)	101 (8.69, 2)
hGIPR (E288G + E354Q) [c]	0.726 (0.0719, 2)	97.1 (2.09, 2)	0.572 (0.0575, 2)	98.1 (2.92, 2)
hGIPR (R101H) [c]	0.125 (n/a, 1)	100 (n/a, 1)	0.259 (n/a, 1)	97.1 (n/a, 1)

Supplementary Table 6. Descriptions of phenotypes (BMI, HbA1c and T2D) used in association analyses in UK Biobank (adapted from Gardner et al. (80)). This table is provided in Excel format, uploaded separately.
